# Prognostic evaluation using nutrition-inflammation biomarkers from routine blood tests in metastatic breast cancer: a Boruta algorithm-optimized feature selection study

**DOI:** 10.3389/fonc.2026.1834427

**Published:** 2026-05-21

**Authors:** Wen-xiong Nong, Sheng-kai Huang, Wen-hai Zhang, Yang Tan, Zhi-dong Wu, Wan-wang Liang, Rui-zheng Wu

**Affiliations:** 1 Department of Breast Surgery, Eighth Affiliated Hospital of Guangxi Medical University, Guigang City People’s Hospital, Guigang, Guangxi, China; 2 Department of Breast Surgery, Guangxi Medical University Tumor Hospital, Nanning, Guangxi, China; 3 Department of Pathology, Liuzhou People’s Hospital/Liuzhou People’s Hospital affiliated to Guangxi Medical University, Liuzhou, Guangxi, China

**Keywords:** blood biomarkers, inflammatory indices, metastatic breast cancer, nutritional indices, prognosis

## Abstract

**Objective:**

To evaluate and compare the prognostic value of multiple nutrition- and inflammation-related indices derived from routine blood tests in patients with metastatic breast cancer, and to identify the most clinically applicable indicators.

**Methods:**

This retrospective study analyzed data from 163 newly diagnosed breast cancer patients with a single distant metastasis. Seventeen nutrition- and inflammation-related indices were assessed, including ALI, NLR, dNLR, SII, SIRI, PLR, MLR, NMLR, PNI, GLR, AGR, GNRI, mGNRI, TP, PA, TRF, and ALB. Kaplan–Meier survival analysis and Cox regression models were used to evaluate the associations between these indices and overall survival (OS). The predictive performance of each index was assessed using the concordance index (C-index), and the Boruta algorithm was applied to identify key variables. Restricted cubic spline (RCS) regression was performed to explore potential nonlinear relationships between indices and OS, and subgroup analyses were conducted to examine index performance across different clinical characteristics.

**Results:**

Multivariate Cox regression analysis identified MLR, SIRI, ALI, AGR, and PA as independent predictors of OS in patients with metastatic breast cancer. Patients with MLR ≥ 0.33 had a 3.94-fold increased risk of death, and those with SIRI ≥ 1.70 had a 3.32-fold increased risk. In contrast, patients with ALI ≥ 53.99, AGR ≥ 1.11, and PA ≥ 181 had 73%, 76%, and 77% reductions in mortality risk, respectively. Inflammation-related indices demonstrated stronger predictive value for short-term (1-year) outcomes, whereas nutrition-related indices were more effective in predicting medium- to long-term (3–5 years) survival. ALI and MLR showed consistent prognostic performance across all clinical subgroups, while SIRI, AGR, and PA exhibited significant interactions in specific subgroups.

**Conclusion:**

Nutrition- and inflammation-related indices derived from routine blood tests—particularly MLR, SIRI, ALI, AGR, and PA—serve as effective tools for prognostic assessment in metastatic breast cancer. Among these, ALI and MLR have universal applicability across patient subgroups, offering clinicians valuable guidance for optimizing patient management strategies and improving survival and quality of life.

## Background

As of 2022, breast cancer remains the most common malignant tumor among women worldwide, with both its incidence and mortality rates ranking first among all female malignancies (1). Despite significant advances in early diagnosis and treatment, a considerable proportion of patients present with distant metastasis at initial diagnosis or develop metastasis after treatment (2), which severely impacts their prognosis and quality of life. The prognosis of patients with metastatic breast cancer varies significantly (3), highlighting the urgent need to establish simple and effective biomarkers to identify high-risk individuals and guide personalized treatment strategies.

In recent years, numerous studies have demonstrated that tumor development and progression are closely related to the nutritional status of the body and systemic inflammatory response (4, 5). Malignant tumors can lead to malnutrition through various mechanisms, including reduced appetite, metabolic disorders, and increased consumption of nutrients (6). At the same time, chronic inflammation within the tumor microenvironment is considered a key factor promoting tumor progression and metastasis (7). Therefore, nutrition and inflammation-related indicators based on routine blood tests could provide a simple, feasible, and cost-effective method for assessing the prognosis of cancer patients. Several studies have reported the prognostic value of different nutrition- and inflammation-related indices in various solid tumors. Among these, low serum albumin (ALB) levels, high neutrophil count, low prognostic nutritional index (PNI), high neutrophil-to-lymphocyte ratio (NLR), and high platelet-to-lymphocyte ratio (PLR) are associated with poorer overall survival (OS) (8–10). The albumin-to-globulin ratio (AGR) (11), preoperative glucose-to-lymphocyte ratio (GLR) (12), and geriatric nutritional risk index (GNRI) (13) are independent predictors of long-term survival in breast cancer patients. Derived markers such as the neutrophil-to-lymphocyte ratio (dNLR), systemic inflammation response index (SIRI), and systemic immune inflammation index (SII) not only predict breast cancer prognosis but are also related to cancer staging (14–16). Furthermore, newly proposed nutrition/inflammation markers, such as the advanced lung cancer inflammation index (ALI), monocyte-to-lymphocyte ratio (MLR), and modified geriatric nutritional risk index (mGNRI), have not been sufficiently studied in relation to metastatic breast cancer.

Although research has confirmed that some of these nutrition/inflammation-related indicators have prognostic value in predicting survival in breast cancer patients, studies specifically focusing on metastatic breast cancer remain insufficient. This is particularly critical because metastatic breast cancer carries a higher risk of malnutrition and systemic inflammation. Therefore, it is necessary to identify the best prognostic factors for patients with metastatic breast cancer. In this study, we assessed and compared the predictive and prognostic roles of 17 nutrition/inflammation biomarkers related to overall survival (OS) in patients with metastatic breast cancer. We aimed to identify the most clinically valuable indicators and explore their applicability in different clinical subgroups, providing reference for clinical management and prognosis evaluation of patients with metastatic breast cancer.

## Materials and methods

### Participants

**Figure 1:**
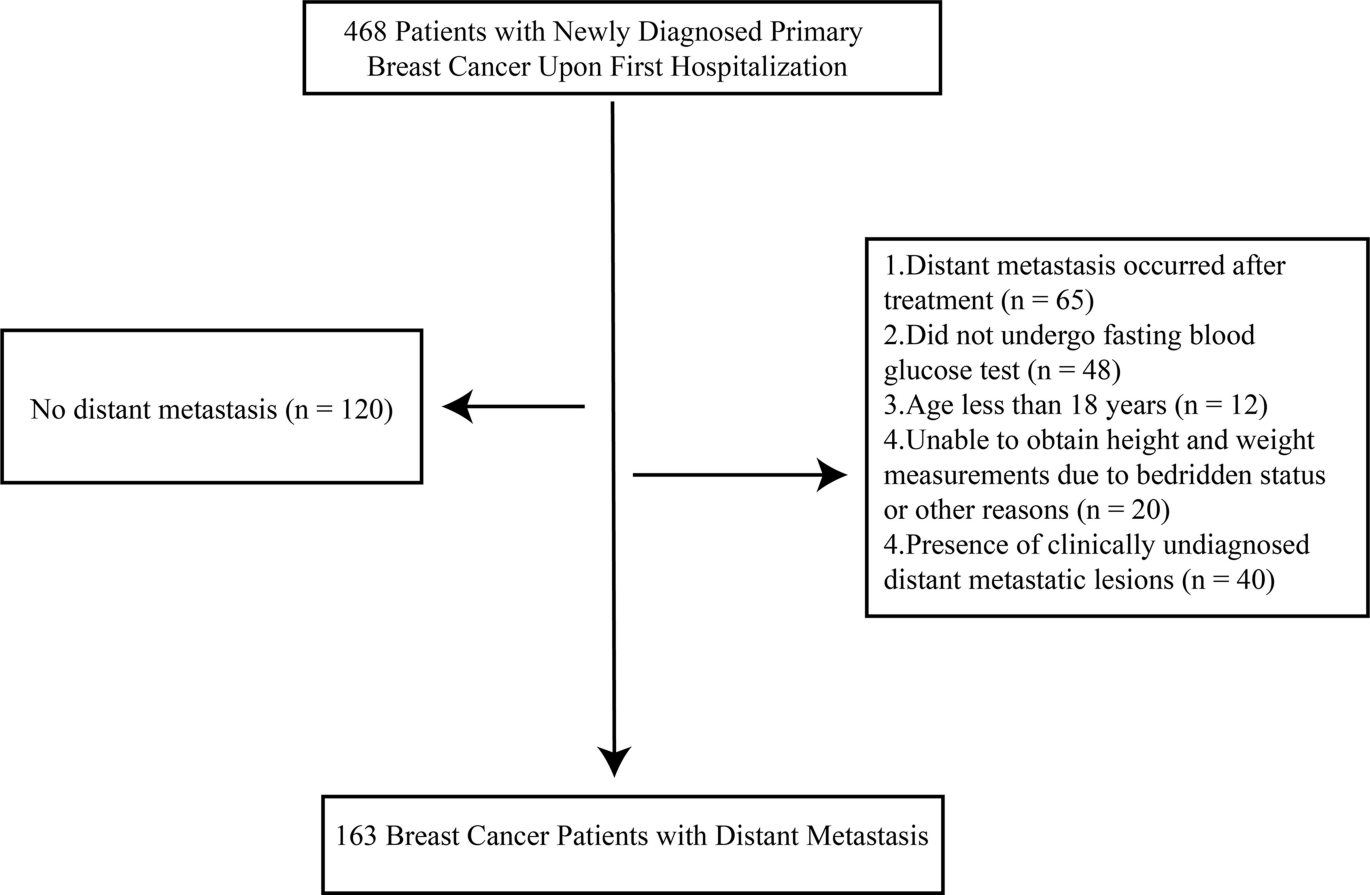
Study flowchart of patient selection.

This study is a retrospective cohort study, involving breast cancer patients who were first admitted to the Affiliated Cancer Hospital of Guangxi Medical University between January 2018 and December 2022 (Ethics Review No.: KY2023868). Inclusion criteria were: (1) Pathologically confirmed primary breast cancer at the initial diagnosis, with or without axillary lymph node metastasis, but with distant metastasis limited to a single lesion (including bone, liver, or lung metastasis); (2) All patients had completed clinical blood biomarker testing before any treatment interventions (radiotherapy, chemotherapy, or surgery); (3) Exclusion of patients with growth and developmental abnormalities (e.g., rickets, acromegaly/gigantism), acute or chronic kidney disease/inflammation, liver dysfunction, or abnormal blood routine history; (4) No other primary malignant tumors. Exclusion criteria were: (1) Distant metastasis occurring after treatment; (2) Lack of liver function or blood routine test data; (3) Age < 18 years; (4) Inability to obtain height and weight data; (5) Indeterminate diagnosis of metastatic lesions. A total of 163 cases were included and followed up regularly via phone, email, and outpatient visits, with the specific inclusion process shown in **Figure 1**. Given the retrospective design using previously collected and de-identified routine clinical data, the requirement for written informed consent was waived by the institutional review board.

### Baseline data collection and measurements of nutrition/inflammation-based indicators in routine blood tests

All patients underwent pathological examination before treatment, including biopsy or partial excisional biopsy, to confirm the diagnosis of invasive breast cancer. Primary breast cancer with distant metastasis was diagnosed through whole-body computed tomography (CT), magnetic resonance imaging (MRI), positron emission tomography-computed tomography (PET-CT), or pathological biopsy. Baseline demographic information, clinical pathological data, and physical measurement data at the time of initial diagnosis were extracted from the patients’ medical records.

All clinical biomarker tests were conducted following standardized protocols to ensure consistency of results across the entire cohort. Blood samples were collected within 24 hours of admission after fasting for at least 9 hours. Whole blood cell counts were measured using an automatic blood analyzer (Mindray CAL8000plus, Shenzhen, China). Liver function indicators, including total protein (TP), prealbumin (PA), transferrin (TRF), albumin (ALB), and fasting blood glucose, were detected using an automatic biochemical analyzer (Siemens ADVIA Chemistry XPT). To minimize batch-to-batch variation, all tests were performed in the same laboratory, adhering strictly to the quality control system established by the National Clinical Laboratory Center (NCCL), with regular instrument calibration and internal quality control.

The biomarkers measured in this study included total protein (TP), prealbumin (PA), transferrin (TRF), albumin (ALB), fasting blood glucose, globulin, and counts of lymphocytes, white blood cells, monocytes, neutrophils, and platelets. Additionally, this study analyzed 17 nutrition/inflammation-related indicators, including the Advanced Lung Cancer Inflammation Index (ALI), neutrophil-to-lymphocyte ratio (NLR), derived neutrophil-to-lymphocyte ratio (dNLR), systemic immune-inflammation index (SII), systemic inflammation response index (SIRI), platelet-to-lymphocyte ratio (PLR), monocyte-to-lymphocyte ratio (MLR), neutrophil-to-monocyte and lymphocyte ratio (NMLR), prognostic nutritional index (PNI), glucose-to-lymphocyte ratio (GLR), albumin-to-globulin ratio (AGR), geriatric nutritional risk index (GNRI), modified geriatric nutritional risk index (mGNRI), total protein (TP), prealbumin (PA), transferrin (TRF), and albumin (ALB). The calculation methods for these nutrition- and inflammation-related indices are detailed in **Supplementary Table 1**.

### Statistical analysis

All statistical analyses were performed using R software (version 4.3.2), including R packages: tableone, survminer, survival, survivalROC, pec, rms, car, ggplot2, reshape2, corrplot, and dplyr. Continuous variables were presented as means ± standard deviations or medians (interquartile ranges) based on their distribution characteristics, while categorical variables were expressed as frequencies (percentages). The normality of the data was assessed using the Shapiro-Wilk test. For continuous variables that followed a normal distribution, independent sample t-tests were used for group comparisons, while Mann-Whitney U tests were applied for non-normally distributed continuous variables. For categorical variables, Fisher’s exact test was used when the expected frequency was less than 5, and the chi-square test was used when the expected frequency was 5 or greater. The optimal cutoff points for continuous nutrition- and inflammation-related indices were determined using the maximum chi-square statistics for dichotomization. Kaplan–Meier curves were used to estimate overall survival (OS), and differences were analyzed using two-sided log-rank tests. Cox proportional hazards regression models were employed to analyze the relationship between nutrition- and inflammation-related indices and patient prognosis. Three models were constructed for each indicator: Model 1 was the unadjusted raw model; Model 2 adjusted for demographic and clinical characteristics such as age, education level, insurance status, hypertension, diabetes, marital status, residency, and menstrual status; Model 3 further adjusted for pathological type, histological grade, molecular subtype, surgery, radiotherapy, and chemotherapy. Detailed treatment-related variables, including treatment lines, endocrine therapy, targeted therapy, anti-HER2 therapy, treatment sequence, and treatment response, were not completely available in the retrospective dataset and therefore could not be included in the multivariable adjustment. The predictive ability of each indicator for OS at 1, 3, and 5 years was evaluated using the C-index. Spearman’s correlation analysis was used to assess correlations among nutrition- and inflammation-related indicators, and a correlation heatmap was generated. The Boruta algorithm was used as an auxiliary feature selection method to identify the most relevant indicators from the full set of candidate biomarkers. Because many of these indicators are mathematically or biologically correlated, Boruta was applied to reduce the dimensionality of the biomarker panel and to prioritize variables with greater prognostic relevance. Boruta was not used as a substitute for survival modeling. Instead, variables identified as important by Boruta were subsequently incorporated into Cox proportional hazards regression analyses to estimate their associations with overall survival.

After Boruta feature selection, the selected indicators were further evaluated using multivariable Cox regression, RCS analysis, time-dependent discrimination analysis, subgroup analysis, and bootstrap internal validation. To assess multicollinearity among the Boruta-selected variables, variance inflation factor (VIF) analysis was performed. A VIF value below 5 was considered to indicate no substantial multicollinearity. To further evaluate the stability of the selected variables and reduce optimism, bootstrap internal validation with 1,000 resamples was performed. To explore potential nonlinear relationships between nutrition- and inflammation-related indices and OS, RCS analysis was used. RCS analysis typically uses 3–5 knots, with the knot positions set at the 10th, 50th, and 90th percentiles of the distribution of the indicators, to flexibly display the nonlinear relationship between continuous changes in the indicator and the risk ratio. This method avoids information loss associated with traditional categorization and helps identify potential threshold effects and risk turning points. Additionally, time-dependent C-index was used to evaluate the changing trend of the predictive ability of each indicator over time.Subgroup analyses were conducted to further determine the applicability of important indicators across different clinical subgroups, including age (≤50 years and >50 years), histological grade (Grade 2 and Grade 3), molecular subtype (e.g., HR+/HER2-, HR-/HER2+), metastatic site (bone, liver, lung), and whether patients underwent surgery, radiotherapy, or chemotherapy. The results of the subgroup analyses were presented in forest plots to clearly show the predictive effects of each indicator within various clinical characteristic subgroups. To provide a clinically actionable output, a simplified risk stratification system was developed based on MLR and ALI, the two indicators that showed broad applicability across clinical subgroups. Patients were classified into three risk groups according to the number of adverse biomarker profiles: low risk, defined as MLR < 0.33 and ALI ≥ 53.99; intermediate risk, defined as either MLR ≥ 0.33 or ALI < 53.99; and high risk, defined as MLR ≥ 0.33 and ALI < 53.99. Kaplan–Meier survival analysis and Cox regression were used to compare overall survival among the three risk groups. As an exploratory analysis, we additionally constructed a combined Cox-based prognostic model using the five key indicators selected by the Boruta algorithm, including MLR, SIRI, ALI, AGR, and PA. A linear risk score was calculated for each patient based on the regression coefficients from the multivariable Cox model. The predictive performance of this combined model for 12-, 36-, and 60-month overall survival was evaluated using time-dependent receiver operating characteristic analysis. The purpose of this exploratory analysis was to examine whether integrating the selected indicators provided incremental predictive value over individual biomarkers. To evaluate the internal robustness of the selected biomarkers and to reduce optimism related to data-driven cutoff selection, internal validation was performed using bootstrap resampling. A total of 1,000 bootstrap resamples were generated. For each bootstrap sample, the Cox model was refitted, and predictive performance was evaluated both in the bootstrap sample and in the original cohort. The average difference between bootstrap-sample performance and test performance in the original cohort was used to estimate optimism. Optimism-corrected C-index values and time-dependent area under the curve (AUC) values at 12, 36, and 60 months were then calculated. This validation procedure was performed for the exploratory combined Cox model and for the five key individual biomarkers selected by the Boruta algorithm.

All statistical tests were two-sided, with a P value of <0.05 considered statistically significant. Data visualization was mainly performed using the ggplot2 package (version 3.4.1), and all charts were standardized to ensure consistency in the visualization results. Sensitivity analyses, including different cutoff strategies and repeated analyses after data standardization, were performed to ensure the robustness of the findings.

## Results

### Characteristics of the patients

**Table 1** presents the baseline demographic and clinical characteristics of the 163 breast cancer patients with distant metastasis included in the study. The average age of the patients was 51 ± 11 years, with 69.3% having medical insurance. The prevalence of hypertension and diabetes was 17.2% and 4.9%, respectively. Regarding marital status, the majority of patients were married (91.4%). In terms of education level, 20.2% had completed primary school, 26.4% had completed middle school, 8.0% had completed high school, 13.5% had completed undergraduate studies, and 31.9% had other education levels. Among the patients, 54.0% lived in rural areas, and 55.2% were postmenopausal. Pathologically, 93.3% of patients were diagnosed with invasive ductal carcinoma, and 78.5% had a histological grade of 2. In terms of molecular subtypes, the most common was HR+/HER2- (54.6%), followed by HR+/HER2+ (21.5%), HR-/HER2+ (17.8%), and HR-/HER2- (6.1%). For the tumor T stage, the most common was T4 (57.1%), and the most frequent lymph node N stage was N1 (40.5%). The most common sites of distant metastasis were bone (57.7%), lung (25.2%), and liver (17.2%). In terms of treatment, 42.3% of patients underwent surgery, 14.7% received radiotherapy, and 67.5% received chemotherapy. Based on the baseline laboratory results, most routine blood parameters were within clinically plausible ranges, while the derived nutrition- and inflammation-related indices showed variable distributions across the cohort.

**Table 1:** Baseline demographic, clinicopathological, and laboratory characteristics of the included patients.

Characteristic	N = 163
**Age (years),Mean ± SD**	51 ± 11
Insurance, n (%)	
No	50 (30.7%)
Yes	113 (69.3%)
Hypertension, n (%)	
No	135 (82.8%)
Yes	28 (17.2%)
Diabetes, n (%)	
No	155 (95.1%)
Yes	8 (4.9%)
Marital status, n (%)	
Married	149 (91.4%)
Unmarried	6 (3.7%)
Widow	6 (3.7%)
Divorced	2 (1.2%)
Education, n (%)	
Primary School	33 (20.2%)
Middle School	43 (26.4%)
High School	13 (8.0%)
Undergraduate	22 (13.5%)
Others	52 (31.9%)
Location, n (%)	
Village	88 (54.0%)
City	75 (46.0%)
Menstrual status, n (%)	
Postmenopausal	90 (55.2%)
Premenopausal	73 (44.8%)
Pathological type, n (%)	
Invasive ductal carcinoma	152 (93.3%)
Invasive lobular carcinoma	6 (3.7%)
Others	5 (3.1%)
Histological grade, n (%)	
2	128 (78.5%)
3	35 (21.5%)
Subtype, n (%)	
HR+/HER2-	89 (54.6%)
HR+/HER2+	35 (21.5%)
HR-/HER2-	10 (6.1%)
HR-/HER2+	29 (17.8%)
T stage, n (%)	
T1	8 (4.9%)
T2	46 (28.2%)
T3	16 (9.8%)
T4	93 (57.1%)
N stage, n (%)	
N0	15 (9.2%)
N1	66 (40.5%)
N2	40 (24.5%)
N3	42 (25.8%)
Metastatic site, n (%)	
Bone	94 (57.7%)
Liver	28 (17.2%)
Lung	41 (25.2%)
Surgery, n (%)	
No	94 (57.7%)
Yes	69 (42.3%)
Radiotherapy, n (%)	
No	139 (85.3%)
Yes	24 (14.7%)
Chemotherapy, n (%)	
No	53 (32.5%)
Yes	110 (67.5%)
**Ki-67,Median (IQR)**	0.35 (0.20, 0.50)
Globulin (g/L), Median (IQR)	29.8 (27.1, 34.0)
Glucose (mmol/L), Median (IQR)	4.82 (4.42, 5.46)
**Transferrin (g/L), Median (IQR)**	2.40 (2.04, 2.65)
**Prealbumin (mg/L), Median (IQR)**	223 (184, 260)
**White Blood Cell (10^9/L), Median (IQR)**	6.53 (5.46, 7.70)
**Platelet (10^9/L), Mean ± SD**	277 ± 83
**Monocyte (10^9/L), Median (IQR)**	0.41 (0.34, 0.52)
**Total Protein (g/L), Mean ± SD**	69 ± 7
**Albumin (g/L), Mean ± SD**	38.8 ± 4.4
**Neutrophil (10^9/L), Median (IQR)**	4.15 (3.23, 5.17)
**Lymphocyte (10^9/L), Median (IQR)**	1.73 (1.42, 2.13)
**BMI, Mean ± SD**	22.9 ± 3.3
**NLR, Median (IQR)**	2.36 (1.73, 3.33)
**ALI, Median (IQR)**	38 (26, 54)
**dNLR, Median (IQR)**	0.87 (0.85, 0.90)
**SII, Median (IQR)**	618 (412, 981)
**SIRI, Median (IQR)**	0.98 (0.62, 1.43)
**PLR, Median (IQR)**	150 (118, 192)
**MLR, Median (IQR)**	0.23 (0.18, 0.31)
**NMLR, Median (IQR)**	2.57 (1.88, 3.56)
**PNI, Mean ± SD**	48.0 ± 5.7
**GLR, Median (IQR)**	2.88 (2.24, 3.52)
**AGR, Median (IQR)**	1.29 (1.12, 1.47)
**GNRI, Mean ± SD**	103 ± 9
**mGNRI, Median (IQR)**	99 (94, 103)

NLR, neutrophil-to-lymphocyte Ratio; dNLR, derived neutrophil-to-lymphocyte ratio; SII, systemic immune-inflammation index; SIRI, systemic inflammatory response index; PLR, platelet-to-lymphocyte ratio; MLR, monocyte-to-lymphocyte ratio; NMLR, neutrophil-to-monocyte-to-lymphocyte ratio; PNI, prognostic nutritional index; GLR, glucose-to-lymphocyte ratio; AGR, albumin to globulin ratio; GNRI, geriatric nutritional risk index; mGNRI, modified geriatric nutritional risk index; ALI, advanced lung cancer inflammation index; BMI, body mass index. Bold text indicates category headings or variable groups within the table and does not denote statistical significance.

### Nutrition- and inflammation-related indices associated with OS in metastatic breast cancer

**Figure 2:**
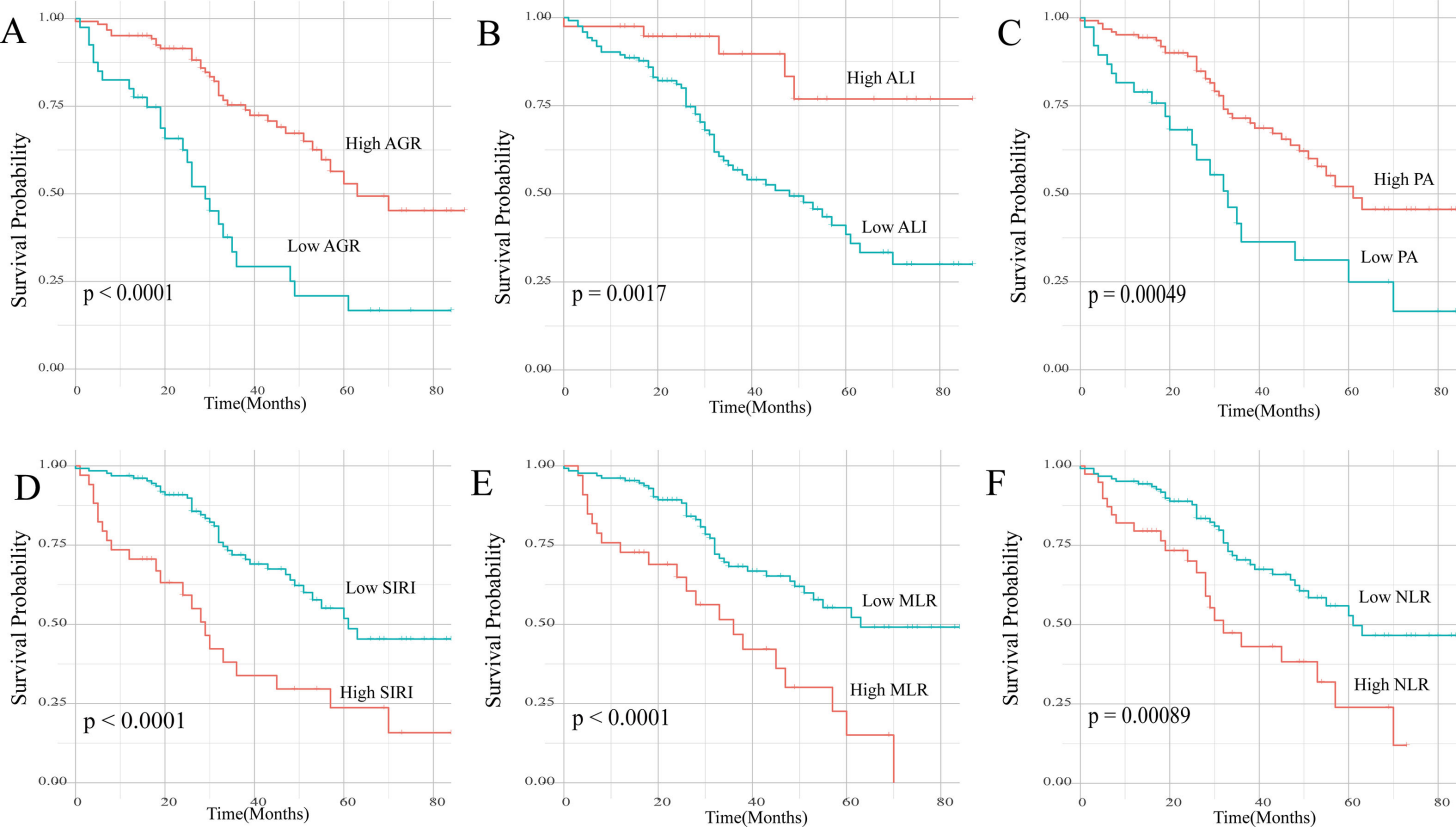
Kaplan–Meier survival curves for overall survival in patients with metastatic breast cancer stratified by AGR **(A)**, ALI **(B)**, PA **(C)**, SIRI **(D)**, MLR **(E)**, and NLR **(F)**. AGR, albumin-to-globulin ratio; ALI, advanced lung cancer inflammation index; PA, prealbumin; SIRI, systemic inflammatory response index; MLR, monocyte-to-lymphocyte ratio; NLR, neutrophil-to-lymphocyte ratio.

The median follow-up time for the patients included in the study was 43 months, and the median overall survival (OS) was 57 months. The optimal cutoff values for the 17 inflammation and nutrition-related indicators were determined using the maximum chi-square statistics, and these cutoff values were as follows: ALI 53.99, NLR 3.35, dNLR 0.90, SII 739.19, SIRI 1.70, PLR 139.41, MLR 0.33, NMLR 3.80, PNI 43.75, GLR 2.38, AGR 1.11, GNRI 96.09, mGNRI 93.37, TP 67.4, PA 181, TRF 2.68, and ALB 39.2. Univariate Cox regression analysis (Model 1) and multivariate analysis adjusting for confounding factors (Models 2 and 3) were used to evaluate the relationship between these indicators and patient prognosis (**Table 2**). In the fully adjusted model (Model 3, adjusted for age, education level, insurance status, hypertension, diabetes, marital status, residency, menopausal status, pathological type, histological grade, molecular subtype, surgery, radiotherapy, and chemotherapy), several inflammation and nutrition indicators were significantly associated with overall survival (OS) in patients with metastatic breast cancer. Among them, MLR, SIRI, AGR, PA, and ALI showed the strongest independent predictive abilities: For MLR, each 1 standard deviation increase in MLR was associated with an 80% increased risk of death (HR 1.80, p<0.001), and patients with MLR ≥ 0.33 had a nearly 4-fold increased risk of death (HR 3.94, p<0.001); for SIRI, each 1 standard deviation increase was associated with a 44% increased risk of death (HR 1.44, p=0.003), and patients with SIRI ≥ 1.70 had a 3.32-fold increased risk of death (HR 3.32, p=0.001); for AGR, each 1 standard deviation increase was associated with a 40% reduction in death risk (HR 0.60, p=0.003), and patients with AGR ≥ 1.11 had a 76% reduced risk of death (HR 0.24, p<0.001); for PA, patients with PA ≥ 181 had a 77% reduced risk of death (HR 0.23, p<0.001); for ALI, each 1 standard deviation increase was associated with a 51% reduction in death risk (HR 0.49, p=0.007), and patients with ALI ≥ 53.99 had a 73% reduced risk of death (HR 0.27, p=0.008).In addition, NLR, SII, PLR, NMLR, GNRI, and TP also showed varying degrees of independent prognostic value, whereas dNLR, PNI, mGNRI, TRF, and ALB were not significantly associated with OS in the fully adjusted model. Kaplan–Meier survival analysis showed that patients with metastatic breast cancer with malnutrition (indicated by lower AGR and lower PA) and higher inflammation levels (indicated by higher SIRI, higher MLR, higher NLR, and lower ALI) had significantly lower OS compared to patients without malnutrition or with normal inflammation levels (**Figure 2**; **Supplementary Figure 1**).

**Table 2:** Univariate and multivariate Cox analysis of seventeen indicators in patients with metastatic breast cancer.

Characteristic	Model 1			Model 2			Model 3		
	HR	95% CI	*P-value*	HR	95% CI	*P-value*	HR	95% CI	*P-value*
ALI									
Per SD (increased)	0.51	0.34, 0.76	0.001	0.46	0.30, 0.72	<0.001	0.49	0.29, 0.82	0.007
< 53.99	—	—		—	—		—	—	
≥ 53.99	0.3	0.13, 0.70	0.005	0.28	0.11, 0.68	0.005	0.27	0.10, 0.71	0.008
NLR									
Per SD (increased)	1.34	1.12, 1.61	0.001	1.46	1.17, 1.83	0.001	1.49	1.12, 1.97	0.006
< 3.35	—	—		—	—		—	—	
≥ 3.35	2.14	1.27, 3.62	0.004	2.38	1.36, 4.18	0.003	1.68	0.87, 3.23	0.12
dNLR									
Per SD (increased)	0.97	0.76, 1.25	0.83	1	0.76, 1.32	0.984	1.08	0.81, 1.43	0.614
< 0.9	—	—		—	—		—	—	
≥ 0.9	1.29	0.75, 2.22	0.365	1.12	0.61, 2.05	0.723	1.51	0.75, 3.06	0.247
SII									
Per SD (increased)	1.31	1.11, 1.54	0.001	1.43	1.15, 1.76	<0.001	1.47	1.11, 1.94	0.008
< 739.19	—	—		—	—		—	—	
≥ 739.19	1.96	1.18, 3.24	0.009	2.03	1.17, 3.55	0.012	1.8	0.91, 3.58	0.093
SIRI									
Per SD (increased)	1.17	1.03, 1.34	0.015	1.22	1.04, 1.43	0.015	1.44	1.14, 1.83	0.003
< 1.7	—	—		—	—		—	—	
≥ 1.7	2.92	1.73, 4.94	<0.001	3.41	1.86, 6.24	<0.001	3.32	1.61, 6.83	0.001
PLR									
Per SD (increased)	1.46	1.15, 1.85	0.002	1.55	1.19, 2.01	<0.001	1.24	0.92, 1.66	0.153
< 139.4	—	—		—	—		—	—	
≥ 139.4	2.27	1.29, 4.02	0.005	2.33	1.24, 4.38	0.008	2.59	1.28, 5.23	0.008
MLR									
Per SD (increased)	1.54	1.31, 1.82	<0.001	1.6	1.32, 1.93	<0.001	1.8	1.35, 2.41	<0.001
< 0.33	—	—		—	—		—	—	
≥ 0.33	2.92	1.72, 4.97	<0.001	3.02	1.73, 5.27	<0.001	3.94	2.00, 7.77	<0.001
NMLR									
Per SD (increased)	1.37	1.14, 1.65	<0.001	1.49	1.19, 1.87	<0.001	1.53	1.15, 2.04	0.004
< 3.8	—	—		—	—		—	—	
≥ 3.8	2.55	1.49, 4.35	<0.001	2.63	1.48, 4.69	0.001	1.97	0.95, 4.07	0.066
PNI									
Per SD (increased)	0.72	0.55, 0.94	0.017	0.73	0.55, 0.97	0.029	0.83	0.60, 1.15	0.264
< 43.75	—	—		—	—		—	—	
≥ 43.75	0.5	0.29, 0.87	0.015	0.51	0.28, 0.93	0.027	0.54	0.26, 1.12	0.099
GLR									
Per SD (increased)	0.92	0.69, 1.23	0.559	0.89	0.66, 1.18	0.409	0.81	0.59, 1.11	0.181
< 2.38	—	—		—	—		—	—	
≥ 2.38	1.47	0.82, 2.63	0.198	1.94	1.03, 3.65	0.039	1.99	1.02, 3.91	0.044
AGR									
Per SD (increased)	0.73	0.57, 0.93	0.011	0.7	0.54, 0.91	0.007	0.6	0.43, 0.84	0.003
< 1.11	—	—		—	—		—	—	
≥ 1.11	0.31	0.18, 0.52	<0.001	0.26	0.15, 0.47	<0.001	0.24	0.12, 0.46	<0.001
GNRI									
Per SD (increased)	0.7	0.53, 0.92	0.012	0.7	0.52, 0.95	0.02	0.69	0.50, 0.96	0.028
< 96.09	—	—		—	—		—	—	
≥ 96.09	0.51	0.29, 0.89	0.018	0.56	0.31, 1.01	0.054	0.39	0.19, 0.81	0.012
mGNRI									
Per SD (increased)	0.74	0.56, 0.97	0.03	0.76	0.57, 1.01	0.057	0.8	0.57, 1.10	0.171
< 93.37	—	—		—	—		—	—	
≥ 93.37	0.73	0.44, 1.21	0.223	0.88	0.51, 1.50	0.636	0.82	0.42, 1.58	0.546
TP									
Per SD (increased)	1.1	0.87, 1.41	0.428	1.14	0.89, 1.46	0.29	1.46	1.07, 1.98	0.016
< 67.4	—	—		—	—		—	—	
≥ 67.4	1.62	0.92, 2.84	0.092	2.01	1.07, 3.75	0.029	3.43	1.63, 7.24	0.001
PA									
Per SD (increased)	0.59	0.36, 0.97	0.038	0.56	0.33, 0.94	0.029	0.58	0.31, 1.10	0.097
< 181	—	—		—	—		—	—	
≥ 181	0.43	0.25, 0.74	0.002	0.34	0.18, 0.63	<0.001	0.23	0.11, 0.49	<0.001
TRF									
Per SD (increased)	0.9	0.68, 1.20	0.49	0.84	0.63, 1.13	0.251	0.87	0.61, 1.24	0.446
< 2.68	—	—		—	—		—	—	
≥ 2.68	0.65	0.36, 1.19	0.163	0.58	0.31, 1.08	0.087	0.49	0.20, 1.16	0.104
ALB									
Per SD (increased)	0.79	0.61, 1.03	0.084	0.82	0.63, 1.09	0.172	0.93	0.68, 1.26	0.637
< 39.2	—	—		—	—		—	—	
≥ 39.2	0.64	0.38, 1.07	0.091	0.68	0.39, 1.19	0.179	0.8	0.42, 1.52	0.504

Model 1 was crude model. Model 2 was adjusted for age, education, insurance, hypertension, diabetes, marital status, location, and menstrual status. Model 3 was adjusted for age, education level, insurance, hypertension, diabetes, marital status, residence, menstrual status, pathological type, histological grade, molecular subtype, surgery, radiotherapy, and chemotherapy. NLR, neutrophil-to-lymphocyte Ratio; dNLR, derived neutrophil-to-lymphocyte ratio; SII, systemic immune-inflammation index; SIRI, systemic inflammatory response index; PLR, platelet-to-lymphocyte ratio; MLR, monocyte-to-lymphocyte ratio; NMLR, neutrophil-to-monocyte-to-lymphocyte ratio; PNI, prognostic nutritional index; GLR, glucose-to-lymphocyte ratio; AGR, albumin to globulin ratio; GNRI,geriatric nutritional risk index; mGNRI, modified geriatric nutritional risk index; ALI, advanced lung cancer inflammation index.

### Screening of important and relevant indicators

**Figure 3:**
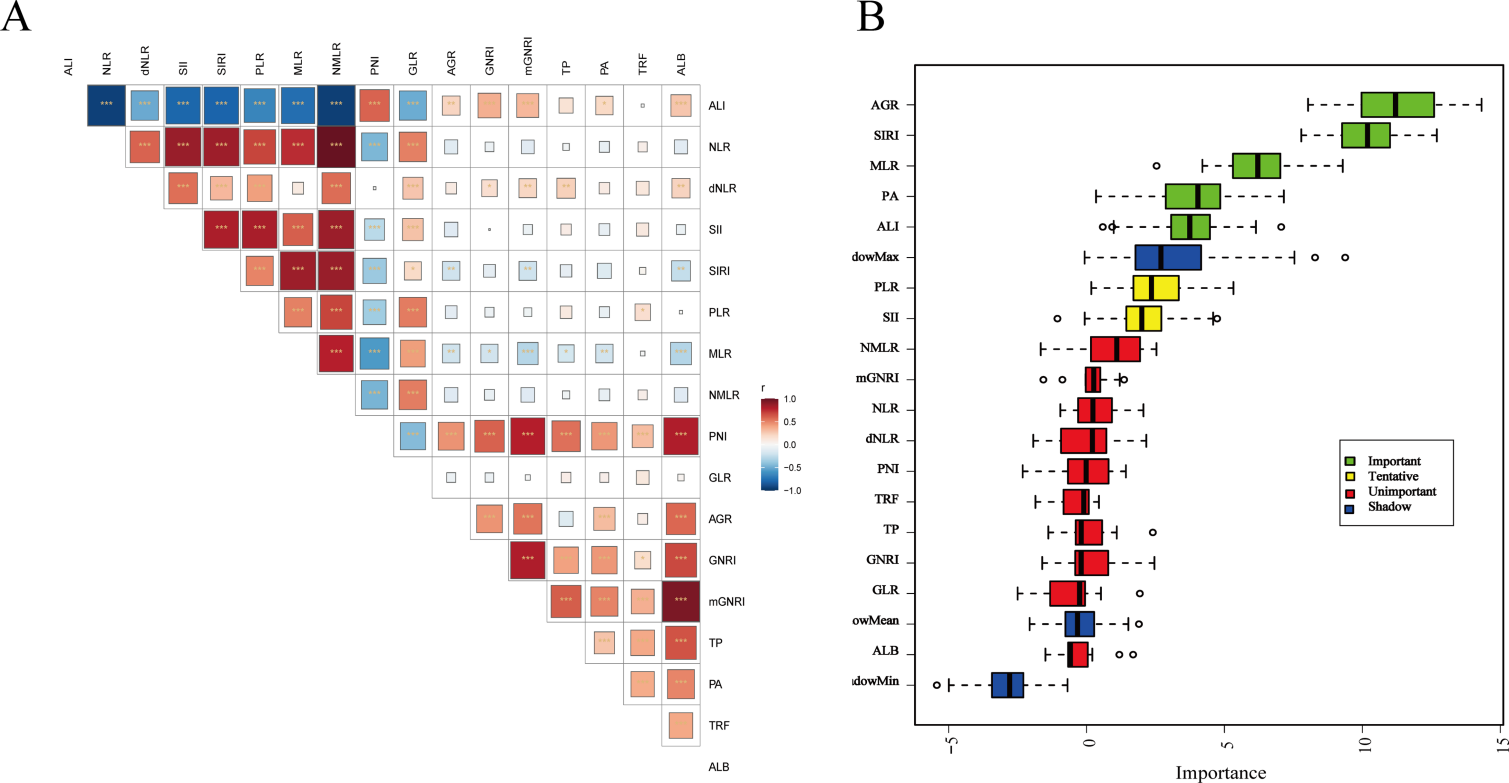
Correlation matrix and Boruta-based variable importance analysis of nutrition- and inflammation-related indicators. **(A)** Heatmap showing pairwise Spearman correlations among the analyzed indicators. The color scale represents the strength and direction of the correlations, and statistical significance is indicated by asterisks. **(B)** Boxplot showing variable importance scores derived from the Boruta algorithm. Variables were classified as important, tentative, unimportant, or shadow variables according to the Boruta selection procedure. NLR, neutrophil-to-lymphocyte ratio; dNLR, derived neutrophil-to-lymphocyte ratio; SII, systemic immune-inflammation index; SIRI, systemic inflammatory response index; PLR, platelet-to-lymphocyte ratio; MLR, monocyte-to-lymphocyte ratio; NMLR, neutrophil-and-monocyte-to-lymphocyte ratio; PNI, prognostic nutritional index; GLR, glucose-to-lymphocyte ratio; AGR, albumin-to-globulin ratio; GNRI, geriatric nutritional risk index; mGNRI, modified geriatric nutritional risk index; ALI, advanced lung cancer inflammation index.

In this study, the C-index was used to evaluate the predictive ability of 17 nutrition and inflammation-related indicators for survival (**Table 3**). When predicting 1-year survival, SIRI performed the best with a C-index of 0.774 (95% CI: 0.603–0.918), followed by SII and MLR, with C-indices of 0.770 (95% CI: 0.646-0.884) and 0.757 (95% CI: 0.586-0.904), respectively. Additionally, NMLR and NLR also showed good predictive performance, with C-indices of 0.747 (95% CI: 0.597-0.881) and 0.743 (95% CI: 0.599-0.878), respectively. When predicting 3-year survival, AGR showed the highest predictive ability, with a C-index of 0.671 (95% CI: 0.573-0.769), followed by SII and ALI, both with C-indices of 0.647 (95% CI: 0.557-0.742). SIRI and MLR also performed well, with C-indices of 0.646 (95% CI: 0.548-0.749) and 0.637 (95% CI: 0.543-0.732), respectively. In predicting 5-year survival, GNRI showed the best predictive value with a C-index of 0.672 (95% CI: 0.547-0.806), followed by MLR (C-index: 0.670, 95% CI: 0.574-0.761) and PNI (C-index: 0.652, 95% CI: 0.533-0.764). ALI also showed good performance with a C-index of 0.650 (95% CI: 0.549-0.746). These results suggest that inflammation-related indicators (such as SIRI, SII, and MLR) perform better in short-term (1-year) prognosis prediction, while nutrition-related indicators (such as AGR, GNRI, and PNI) may be more advantageous for predicting medium- to long-term (3–5 years) survival. Notably, MLR maintained a high predictive ability at all time points.Further, Spearman’s correlation analysis was conducted, and a heatmap was generated to show the correlations between various inflammation and nutrition-related indicators (**Figure 3A**). The results revealed strong positive correlations among inflammation-related indicators (such as NLR, SII, SIRI, PLR, MLR, and NMLR), while ALI showed moderate to strong negative correlations with most inflammation-related indicators. Additionally, nutrition-related indicators (such as AGR, GNRI, PNI, and ALB) exhibited positive correlations with each other and negative correlations with inflammation indicators. To identify the variables most strongly associated with overall survival (OS), the Boruta algorithm was used for variable importance assessment (**Figure 3B**). The Boruta algorithm identified MLR, SIRI, ALI, AGR, and PA as important variables. These variables were not interpreted as an independent prediction model by themselves; instead, they were subsequently evaluated in Cox regression-based survival analyses and additional clinical applicability analyses, including RCS analysis, time-dependent discrimination analysis, subgroup analysis, and bootstrap internal validation.

**Table 3:** The C-index of 17 indicators for overall survival (OS) in patients with metastatic breast cancer.

C-index (95% CI)			
Indicators	First year	Third year	Fifth year
ALI	0.730 (0.565, 0.873)	0.647 (0.557, 0.742)	0.650 (0.549, 0.746)
NLR	0.743 (0.599, 0.878)	0.623 (0.519, 0.716)	0.605 (0.507, 0.700)
dNLR	0.501 (0.322, 0.658)	0.432 (0.320, 0.541)	0.534 (0.375, 0.688)
SII	0.770 (0.646, 0.884)	0.654 (0.548, 0.755)	0.579 (0.472, 0.687)
SIRI	0.774 (0.603, 0.918)	0.646 (0.548, 0.749)	0.608 (0.509, 0.705)
PLR	0.741 (0.602, 0.855)	0.635 (0.530, 0.736)	0.625 (0.517, 0.731)
MLR	0.757 (0.586, 0.904)	0.637 (0.543, 0.732)	0.670 (0.574, 0.761)
NMLR	0.747 (0.597, 0.881)	0.624 (0.527, 0.724)	0.613 (0.516, 0.707)
PNI	0.687 (0.481, 0.854)	0.588 (0.481, 0.694)	0.652 (0.533, 0.764)
GLR	0.537 (0.359, 0.705)	0.511 (0.414, 0.608)	0.573 (0.475, 0.669)
AGR	0.693 (0.532, 0.851)	0.671 (0.573, 0.769)	0.621 (0.516, 0.723)
GNRI	0.560 (0.358, 0.742)	0.567 (0.467, 0.662)	0.672 (0.547, 0.806)
mGNRI	0.655 (0.457, 0.846)	0.553 (0.442, 0.663)	0.618 (0.492, 0.755)
TP	0.507 (0.336, 0.678)	0.584 (0.485, 0.684)	0.487 (0.360, 0.610)
PA	0.669 (0.476, 0.836)	0.616 (0.491, 0.733)	0.607 (0.488, 0.726)
TRF	0.684 (0.551, 0.808)	0.493 (0.366, 0.621)	0.542 (0.377, 0.695)
ALB	0.626 (0.426, 0.816)	0.558 (0.444, 0.656)	0.586 (0.460, 0.716)

NLR, neutrophil-to-lymphocyte Ratio; dNLR, derived neutrophil-to-lymphocyte ratio; SII, systemic immune-inflammation index; SIRI, systemic inflammatory response index; PLR, platelet-to-lymphocyte ratio; MLR, monocyte-to-lymphocyte ratio; NMLR, neutrophil-to-monocyte-to-lymphocyte ratio; PNI, prognostic nutritional index; GLR, glucose-to-lymphocyte ratio; AGR, albumin to globulin ratio; GNRI,geriatric nutritional risk index; mGNRI, modified geriatric nutritional risk index; ALI, advanced lung cancer inflammation index.

To assess potential multicollinearity among the Boruta-selected variables, VIF analysis was performed (**Supplementary Figure 2**). All selected variables had VIF values below 5, indicating no substantial multicollinearity among these indicators. Bootstrap internal validation using 1,000 resamples further suggested acceptable internal robustness of the selected biomarkers after correction for optimism. In an exploratory analysis, we constructed a combined Cox-based prognostic model incorporating the five Boruta-selected indicators: MLR, SIRI, ALI, AGR, and PA. The time-dependent AUC values of the combined model for predicting 12-, 36-, and 60-month overall survival were 0.766, 0.668, and 0.660, respectively. These values did not indicate a clear improvement over the best-performing individual indicators at corresponding time points. Therefore, the subsequent analyses focused on the clinical applicability and subgroup robustness of individual biomarkers rather than emphasizing a more complex combined prediction model. Bootstrap internal validation was performed using 1,000 resamples. For the exploratory combined Cox model including MLR, SIRI, ALI, AGR, and PA, the apparent C-index was 0.685, and the optimism-corrected C-index was 0.668. The apparent time-dependent AUC values at 12, 36, and 60 months were 0.766, 0.668, and 0.660, respectively. After optimism correction, the corresponding AUC values were 0.751, 0.645, and 0.624, respectively. For the individual biomarkers, the optimism-corrected C-index values were 0.645 for MLR, 0.649 for SIRI, 0.633 for ALI, 0.645 for AGR, and 0.613 for PA. The optimism-corrected 12-month AUC values were 0.747 for MLR, 0.753 for SIRI, 0.721 for ALI, 0.667 for AGR, and 0.646 for PA. These findings indicated that the selected biomarkers showed acceptable internal robustness, while the combined model did not demonstrate a substantial incremental improvement after correction for optimism (**Supplementary Table 7**).

### Analysis of clinical applicability of important indicators

**Figure 4:**
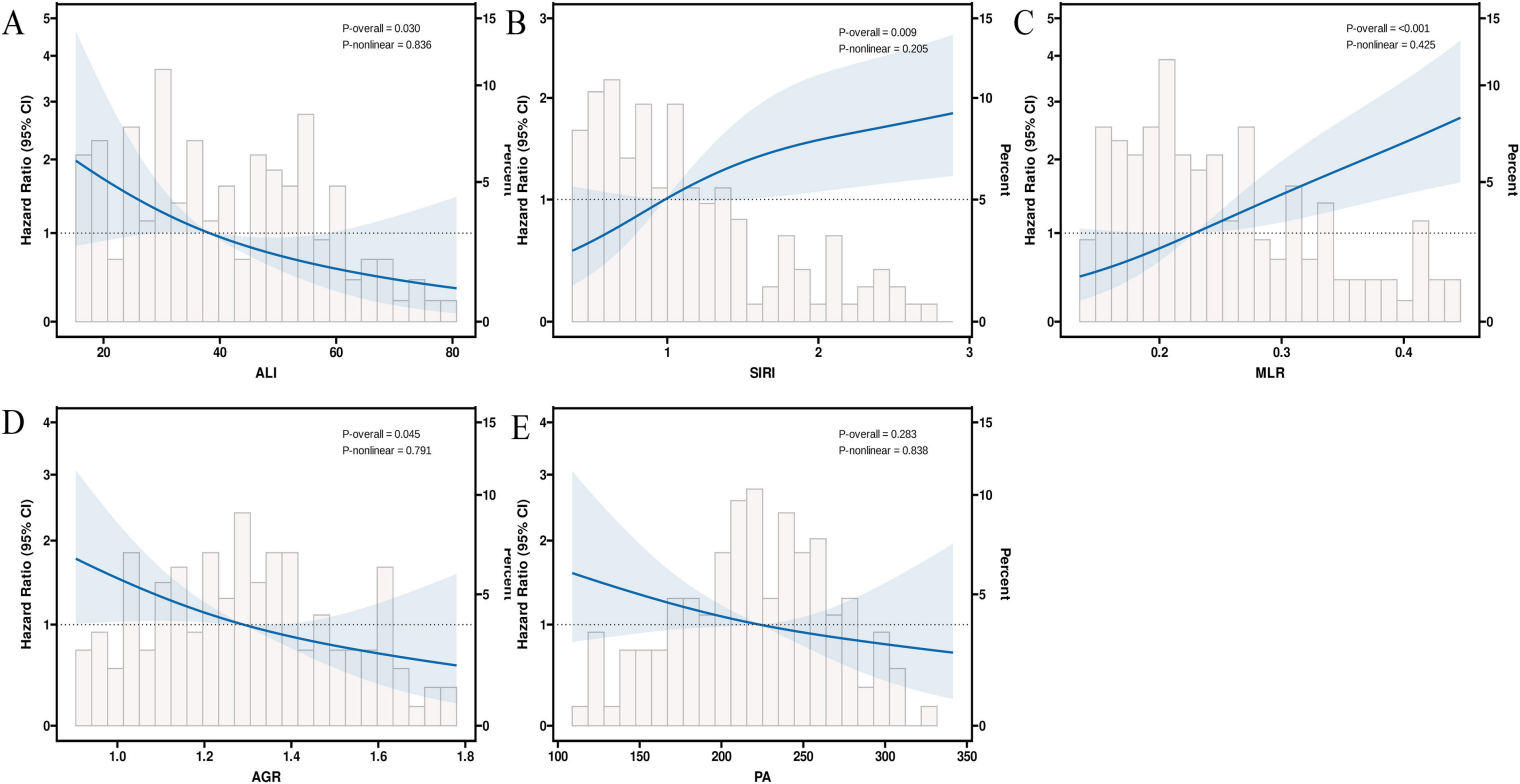
RCS analysis of selected indicators and overall survival. **(A–E)** RCS curves showing the associations of ALI **(A)**, SIRI **(B)**, MLR **(C)**, AGR **(D)**, and PA **(E)** with overall survival. The curves show the estimated hazard ratios with 95% confidence intervals. Histograms indicate the distribution of each indicator. P-overall and P-nonlinear values are shown in each panel. ALI, advanced lung cancer inflammation index; SIRI, systemic inflammatory response index; MLR, monocyte-to-lymphocyte ratio; AGR, albumin-to-globulin ratio; PA, prealbumin.

After variable screening, MLR, SIRI, ALI, AGR, and PA were identified as important indicators significantly associated with overall survival (OS) in patients with metastatic breast cancer. **Supplementary Tables 2**-**S6** present the baseline characteristics of breast cancer patients based on high/low groups of MLR, SIRI, ALI, AGR, and PA. RCS analysis was conducted to explore the nonlinear relationships between these key indicators and overall survival (**Figure 4**). The analysis showed that MLR, SIRI, ALI, AGR, and PA all exhibited linear relationships with survival risk. Specifically, higher values of ALI, AGR, and PA were associated with lower mortality risk, while higher values of MLR and SIRI were associated with higher mortality risk.

**Figure 5:**
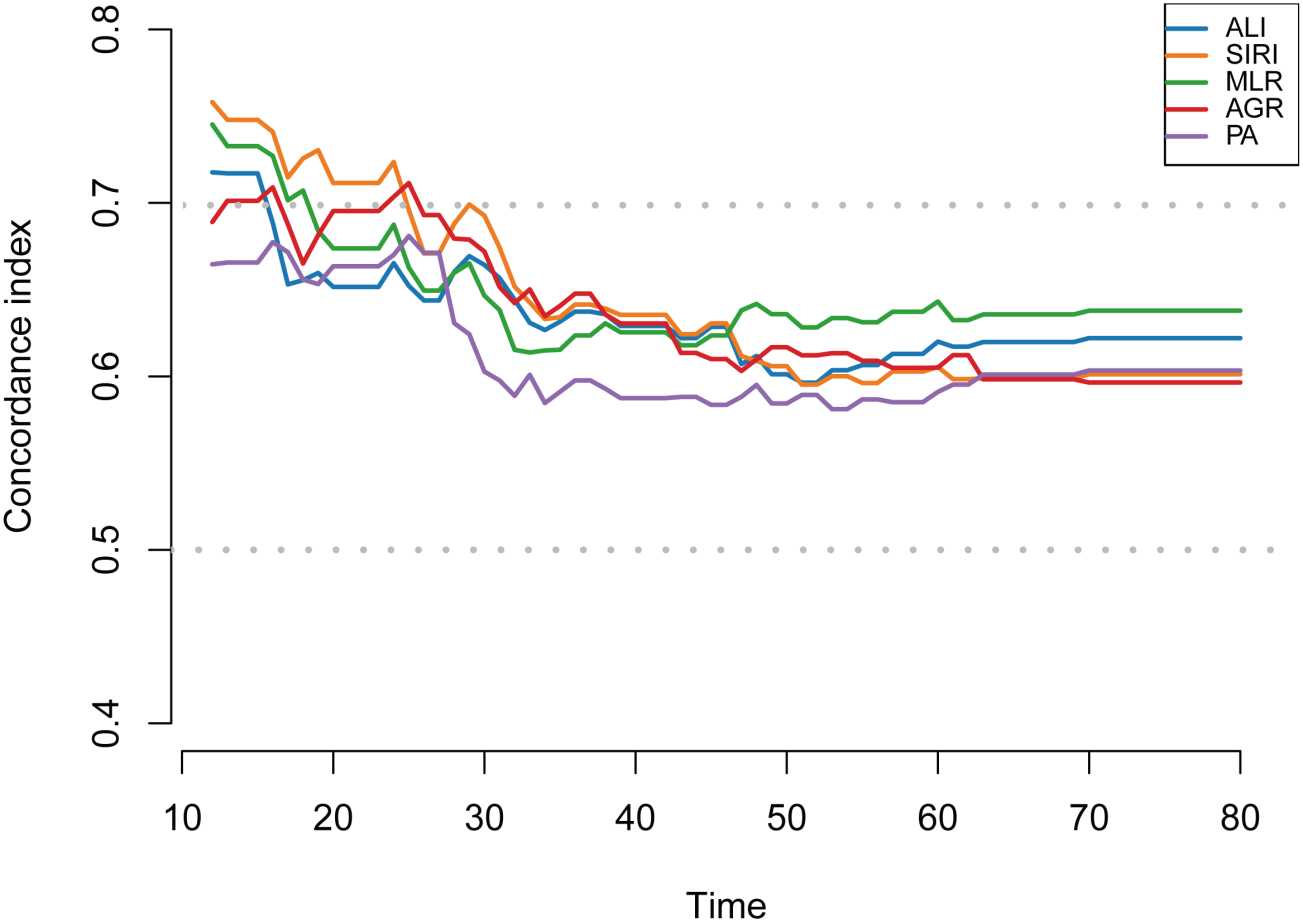
Time-dependent C-index of selected nutrition- and inflammation-related indicators for predicting overall survival in patients with metastatic breast cancer. The figure shows the time-dependent C-index of ALI, SIRI, MLR, AGR, and PA during follow-up. The C-index represents the discriminatory ability of each indicator for overall survival prediction over time. ALI, advanced lung cancer inflammation index; SIRI, systemic inflammatory response index; MLR, monocyte-to-lymphocyte ratio; AGR, albumin-to-globulin ratio; PA, prealbumin.

Additionally, a time-dependent C-index was used to assess the dynamic predictive performance of MLR, SIRI, ALI, AGR, and PA for overall survival in patients with metastatic breast cancer (**Figure 5**). The results showed that during the early follow-up phase (approximately 10–30 months), the predictive ability of each indicator fluctuated, with C-indices ranging from 0.60 to 0.75, with SIRI and MLR demonstrating relatively better predictive ability. In the middle to late follow-up phase (approximately 40–80 months), the predictive ability of each indicator stabilized, with most indicators’ C-indices remaining between 0.60 and 0.65. SIRI and MLR maintained stable predictive performance throughout the follow-up period, while PA consistently exhibited lower predictive ability.

**Figure 6:**
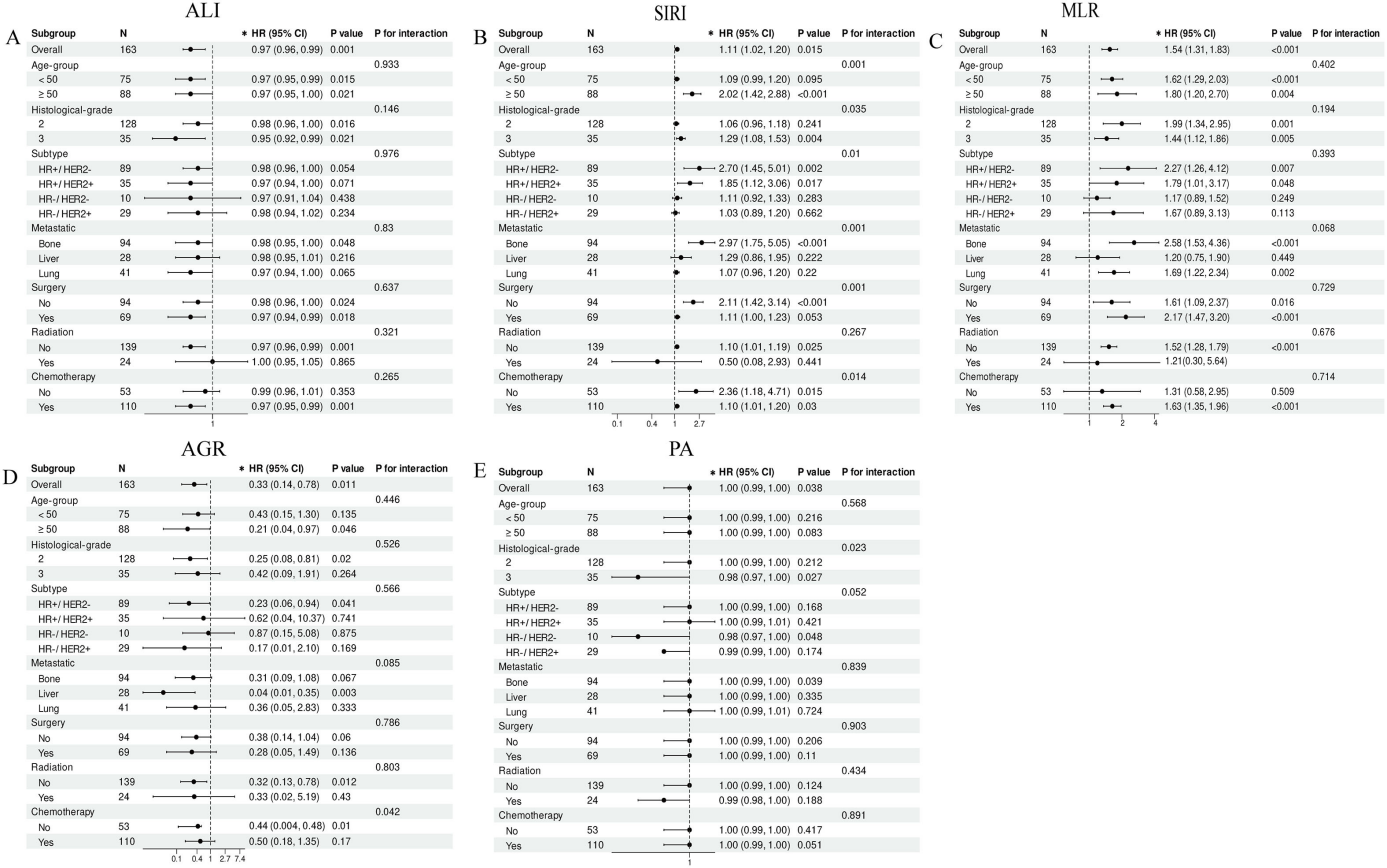
Subgroup analyses of selected indicators for overall survival. **(A–E)** Forest plots showing the hazard ratios and 95% confidence intervals for ALI **(A)**, SIRI **(B)**, MLR **(C)**, AGR **(D)**, and PA **(E)** across predefined clinical subgroups, including age group, histological grade, molecular subtype, metastatic site, surgery, radiotherapy, and chemotherapy. Interaction p-values are shown to assess whether the prognostic associations differed across subgroups. Models were adjusted for age, education level, insurance status, hypertension, diabetes, marital status, residence, menstrual status, pathological type, histological grade, molecular subtype, surgery, radiotherapy, and chemotherapy, as appropriate. ALI, advanced lung cancer inflammation index; SIRI, systemic inflammatory response index; MLR, monocyte-to-lymphocyte ratio; AGR, albumin-to-globulin ratio; PA, prealbumin; HR, hazard ratio; CI, confidence interval.

Moreover, subgroup analyses of MLR, SIRI, ALI, AGR, and PA were conducted to assess the variations in their performance across different patient subgroups (**Figure 6**). The results revealed that ALI and MLR showed good consistency across all subgroups, with no significant interactions (all interaction P-values > 0.05). Based on the broad subgroup applicability of MLR and ALI, we further developed a simplified MLR–ALI risk stratification system. Patients were classified into low-, intermediate-, and high-risk groups according to the combined status of MLR and ALI. Among the 163 patients, 40 were classified as low risk, 92 as intermediate risk, and 31 as high risk. The numbers of death events were 5, 36, and 20 in the low-, intermediate-, and high-risk groups, respectively.

Kaplan–Meier analysis showed a clear stepwise separation of overall survival among the three risk groups (**Supplementary Figure 3**), with patients in the high-risk group having the poorest survival outcomes (log-rank p = 1.98 × 10^-5^). In Cox regression analysis (**Supplementary Table 8**), compared with the low-risk group, the intermediate-risk group had a significantly higher risk of death (HR = 3.081, 95% CI: 1.209–7.853, p = 0.018), while the high-risk group had an even greater risk of death (HR = 7.158, 95% CI: 2.683–19.095, p < 0.001). These findings suggest that the MLR–ALI risk stratification system may provide a simple and clinically interpretable tool for preliminary risk assessment in patients with metastatic breast cancer. Notably, SIRI demonstrated significant interactions across different age groups, histological grades, molecular subtypes, metastatic sites, and whether patients had undergone surgery or chemotherapy. AGR exhibited significant interactions only between the subgroup of patients who received chemotherapy, while PA showed significant interactions only in different histological grade subgroups.

## Discussion

This study retrospectively analyzed 163 newly diagnosed breast cancer patients with a single distant metastasis and systematically evaluated the value of 17 nutrition and inflammation-related indicators based on routine blood tests in predicting patient prognosis. The results indicated that MLR, SIRI, ALI, AGR, and PA are key indicators for predicting overall survival (OS) in patients with metastatic breast cancer, with ALI and MLR showing good predictive stability across various clinical subgroups.

Inflammation plays an important role in the initiation, progression, and metastasis of tumors (17, 18) and has become a major area of cancer research in cancer research. Our study found that inflammation-related indicators, particularly MLR, were significantly associated with overall survival of patients with metastatic breast cancer, consistent with previous studies (19, 20). This observation is also supported by recent large-scale evidence in breast cancer. For example, a large cohort study incorporating inflammation- and nutrition-related biomarkers into a prognostic nomogram showed that blood test-derived indicators could provide additional prognostic information beyond conventional clinicopathological factors (21). Recent meta-analytic evidence has also suggested that systemic inflammatory indices are associated with adverse survival outcomes in breast cancer (22). MLR reflects the balance between circulating monocytes and lymphocytes, with an elevated MLR suggesting increased monocytes and/or decreased lymphocytes. This may be associated with enhanced pro-tumor immune responses in the tumor microenvironment (23). Monocytes are precursor cells for tumor-associated macrophages (TAMs), which secrete various factors that promote tumor angiogenesis, invasion, and immune suppression, thereby facilitating tumor progression and metastasis (24). Decreased lymphocytes indicate impaired anti-tumor immune function, which may lead to immune escape by the tumor (25). Similarly, SIRI integrates the counts of neutrophils, monocytes, and lymphocytes, providing a more comprehensive reflection of the body’s inflammatory and immune status. Our study found that patients with SIRI ≥ 1.70 had a 3.32-fold increased risk of death, suggesting that SIRI could be an important prognostic indicator for patients with metastatic breast cancer. This finding is consistent with recent pooled evidence showing that elevated SIRI is associated with poorer survival and more aggressive clinicopathological features in breast cancer (22). However, unlike most previous studies that included patients across different disease stages, our study focused specifically on newly diagnosed metastatic breast cancer, which may better reflect the prognostic value of systemic inflammation in an advanced-disease setting. Notably, our study revealed that inflammation-related indicators performed better in predicting short-term (1-year) prognosis, which may reflect the active status of the tumor at the time (26). In contrast, nutrition-related indicators such as AGR, GNRI, and PNI performed better in predicting medium- to long-term (3–5 years) survival, potentially reflecting the sustained impact of the patient’s overall nutritional status on long-term survival (27). This pattern is broadly consistent with recent large-scale breast cancer research suggesting that nutritional biomarkers, including albumin-to-globulin ratio and prealbumin, contribute meaningful prognostic information. In our cohort, AGR and PA were also independently associated with overall survival, supporting the clinical relevance of nutritional assessment in metastatic breast cancer. Specifically, ALI, as a composite indicator incorporating BMI, albumin, and NLR, maintained high predictive efficacy across all time points, consistent with previous findings in lung and gastric cancers (28, 29). Serum albumin levels and NLR are also important prognostic factors for OS in breast cancer patients (8, 30). Compared to other indicators, ALI is the only one that encompasses body measurements, nutrition, and inflammation factors. This could be one of the reasons why ALI has better prognostic performance of patients with metastatic breast cancer.

This study also found that the nutritional status of patients has a significant impact on prognosis. AGR and PA, as markers reflecting nutritional status, were closely related to patient survival. Patients with AGR ≥ 1.11 had a 76% lower risk of death, and those with PA ≥ 181 had a 77% lower risk of death. These findings highlight the importance of maintaining good nutritional status in patients with metastatic breast cancer (31). Albumin is a key indicator for assessing nutritional status, while globulin levels are associated with the inflammatory status. Therefore, AGR can simultaneously reflect both the nutritional and inflammatory status of the patient. Prealbumin (PA) has a short half-life (approximately 2 days), making it more sensitive to changes in nutritional status, which may make it an ideal indicator for assessing short-term nutritional status (32).

The restrictive cubic spline curve analysis revealed a linear relationship between MLR, SIRI, ALI, AGR, PA, and survival risk. This suggests that changes in these indicators are associated with continuous changes in prognosis, rather than exhibiting a clear threshold effect. This finding provides a theoretical basis for adjusting treatment strategies based on changes in these indicators in clinical practice.Time-dependent C-index analysis further highlighted the dynamic variation in the predictive ability of each indicator over the follow-up period. Among the indicators, SIRI and MLR exhibited relatively stable predictive effectiveness throughout the follow-up period. This stability is crucial for long-term follow-up management, as it helps clinicians make informed decisions regarding patient care over extended periods.

The results of the subgroup analysis indicated that ALI and MLR demonstrated consistent prognostic value across various clinical subgroups, with no significant interactions observed. This suggests that these two indicators have broad applicability. In contrast, SIRI showed significant interactions in subgroups based on age, histological grade, molecular subtype, metastatic site, and treatment modality. This likely reflects the specificity of SIRI in assessing the prognosis of patients with different clinical characteristics.Several factors could contribute to this interaction phenomenon. First, as an inflammation marker integrating neutrophils, monocytes, and lymphocytes, SIRI shows varying sensitivity to immune state changes in patients of different ages. Age-related immune aging may affect the prognostic value of SIRI (33). Second, breast cancers with different histological grades and molecular subtypes exhibit distinct tumor microenvironment characteristics and patterns of inflammatory infiltration (34), which could lead to differences in the predictive power of SIRI across these subgroups. Furthermore, different metastatic sites such as bone, liver, or lung metastases have unique organ microenvironments and immune response patterns, which could influence the relationship between SIRI, systemic inflammation, and local tumor progression (35). Lastly, the impact of different treatment modalities, such as chemotherapy, radiotherapy, and surgery, on the immune system may alter the prognostic significance of SIRI (36, 37). AGR showed significant interactions only in the chemotherapy subgroup, while PA showed significant interactions only in subgroups based on histological grade. This may be due to several factors: the toxicity of chemotherapy drugs can impair liver function, further affecting albumin synthesis and reinforcing the correlation between AGR and prognosis. Therefore, in chemotherapy patients, AGR may not only reflect underlying nutritional status but also indirectly reflect chemotherapy tolerance and liver function reserve, which could explain the observed interaction (38). High-grade tumors typically have greater invasiveness and higher metabolic demands (39), which may lead to more severe cachexia, making PA a more sensitive short-term nutritional marker with stronger prognostic value in these patients. These findings provide valuable insights for clinicians to select appropriate prognostic indicators for specific patient groups. For instance, AGR may provide more valuable prognostic information for patients undergoing chemotherapy, while PA could have a different prognostic significance in patients with different histological grades. Meanwhile, the use of SIRI should be more individualized, considering factors like patient age, tumor grade, molecular subtype, and metastatic characteristics. In contrast, ALI and MLR, due to their broad applicability, may be more suitable as routine prognostic tools for the general population. Based on the consistent subgroup performance of MLR and ALI, we further proposed a simplified MLR–ALI risk stratification system as a clinically actionable output. This two-marker system showed clear separation of overall survival among low-, intermediate-, and high-risk patients. Compared with a complex nomogram or multivariable risk score, this approach is easier to interpret and implement because it relies on routinely available blood-test-derived indicators and body mass index. Patients with both elevated MLR and reduced ALI may represent a particularly high-risk subgroup requiring closer clinical monitoring, nutritional assessment, and individualized treatment planning. However, this risk stratification system should be considered exploratory and requires external validation before routine clinical application.

Although the construction of combined prognostic models may improve prediction in some settings, our exploratory combined-model analysis did not show a substantial improvement in time-dependent AUC compared with the best-performing individual biomarkers. This finding suggests that, in this cohort, a more complex integrated model may not provide sufficient additional discriminatory value to justify reduced clinical simplicity and interpretability. Therefore, the clinical relevance of the present study lies in identifying parsimonious, routinely available, and robust biomarkers from a large panel of nutrition- and inflammation-related indices. Similar to widely used clinical biomarkers, such indicators may be more suitable for rapid risk stratification and routine follow-up in metastatic breast cancer. To address potential overfitting related to data-driven cutoff selection, we performed bootstrap internal validation. After optimism correction, the selected biomarkers retained generally stable performance, supporting their internal robustness. However, the predictive performance of the exploratory combined model was attenuated after optimism correction and did not show substantial improvement over the best-performing individual biomarkers. This finding supports the clinical rationale of prioritizing simple, interpretable, and routinely available indicators rather than emphasizing a complex prediction model with limited incremental value.

This study has several limitations. First, this was a single-center retrospective study with a relatively small sample size (n = 163), which may limit the robustness and generalizability of the findings. This limitation is particularly relevant to the subgroup analyses, because some subgroup categories included only a small number of patients, resulting in limited statistical power and greater uncertainty in the estimated associations. Therefore, the subgroup results should be interpreted as exploratory and hypothesis-generating rather than confirmatory. Although bootstrap internal validation was performed to reduce optimism, this study still lacks external validation in an independent cohort. Accordingly, the generalizability of the findings, particularly the data-driven cutoff values, should be interpreted with caution. Future multicenter prospective studies with larger sample sizes and independent validation cohorts are needed to confirm the prognostic value and clinical applicability of these indicators. Second, the study only included breast cancer patients with single distant metastasis, and the findings may not be applicable to patients with multiple metastases. Third, although several available clinical and treatment-related variables were adjusted for in the multivariable models, detailed treatment information, including treatment lines, endocrine therapy, targeted therapy, anti-HER2 therapy, treatment sequence, and treatment response, was not completely available. Therefore, residual treatment-related confounding may remain and may have influenced survival outcomes. Finally, this study did not collect longitudinal data on dynamic changes in nutritional and inflammatory indicators during treatment, preventing assessment of how temporal changes in these biomarkers may influence prognosis. Despite these limitations, this study is the first to systematically compare multiple nutrition- and inflammation-related indicators in terms of their prognostic value in metastatic breast cancer. The use of Boruta in this study should be interpreted as a feature prioritization strategy rather than as a standalone prognostic modeling approach. By combining Boruta-based screening with Cox regression, multicollinearity assessment, and bootstrap internal validation, we aimed to identify clinically interpretable biomarkers that were statistically relevant and relatively robust. Importantly, the exploratory combined model did not show substantial incremental predictive value after optimism correction, supporting the clinical rationale of prioritizing simple, accessible, and interpretable individual biomarkers rather than emphasizing a more complex prediction model. The study identified MLR, SIRI, ALI, AGR, and PA as key prognostic indicators. These indicators are derived from routine blood tests, which are easily accessible in clinical practice, cost-effective, and have the potential to serve as valuable tools for prognostic evaluation and individualized treatment decision-making in patients with metastatic breast cancer.Future research should aim to increase the sample size, include multi-center patient data, and conduct prospective validation. Additionally, attention should be paid to the relationship between the dynamic changes of nutrition and inflammation indicators and prognosis, as well as their application value in guiding clinical treatment decisions. Furthermore, integrating these indicators with existing clinical staging and molecular biomarkers to construct a comprehensive prognostic scoring system may further enhance the accuracy of prognostic assessments.

## Conclusion

This study demonstrates that nutrition- and inflammation-related indicators derived from routine blood tests, particularly MLR, SIRI, ALI, AGR, and PA, can serve as effective tools for assessing the prognosis of patients with metastatic breast cancer. Among these, MLR and SIRI are more prominent in short-term (1-year) prognosis, while ALI and MLR have broad applicability across the general population. These simple and easily accessible indicators can assist clinicians in optimizing patient management strategies, ultimately improving survival and quality of life for patients with metastatic breast cancer.

## Data Availability

The original contributions presented in the study are included in the article/**Supplementary Material**. Further inquiries can be directed to the corresponding author.

## References

[1] Bray F Laversanne M Sung H Ferlay J Siegel RL Soerjomataram I . Global cancer statistics 2022: GLOBOCAN estimates of incidence and mortality worldwide for 36 cancers in 185 countries. CA A Cancer J Clin. (2024) 74:229–63. doi: 10.3322/caac.21834. PMID: 38572751

[2] Benitez Fuentes JD Morgan E de Luna Aguilar A Mafra A Shah R Giusti F . Global stage distribution of breast cancer at diagnosis. JAMA Oncol. (2024) 10:71. doi: 10.1001/jamaoncol.2023.4837. PMID: 37943547 PMC10636649

[3] Leone BA Vallejo CT Romero AO Machiavelli MR Pérez JE Leone J . Prognostic impact of metastatic pattern in stage IV breast cancer at initial diagnosis. Breast Cancer Res Treat. (2017) 161:537–48. doi: 10.1007/s10549-016-4066-7. PMID: 27975154

[4] Arends J Bachmann P Baracos V Barthelemy N Bertz H Bozzetti F . ESPEN guidelines on nutrition in cancer patients. Clin Nutr. (2017) 36:11–48. doi: 10.1016/j.clnu.2016.07.015. PMID: 27637832

[5] Diakos CI Charles KA McMillan DC Clarke SJ . Cancer-related inflammation and treatment effectiveness. Lancet Oncol. (2014) 15:e493–503. doi: 10.1016/s1470-2045(14)70263-3. PMID: 25281468

[6] Nishikawa H Goto M Fukunishi S Asai A Nishiguchi S Higuchi K . Cancer cachexia: its mechanism and clinical significance. Ijms. (2021) 22:8491. doi: 10.3390/ijms22168491. PMID: 34445197 PMC8395185

[7] Denk D Greten F . Inflammation: the incubator of the tumor microenvironment. Trends In Cancer. (2022) 8:901–14. doi: 10.1016/j.trecan.2022.07.002. PMID: 35907753

[8] Xiang M Zhang H Tian J Yuan Y Xu Z Chen J . Low serum albumin levels and high neutrophil counts are predictive of a poorer prognosis in patients with metastatic breast cancer. Oncol Lett. (2022) 24:432. doi: 10.3892/ol.2022.13552. PMID: 36311691 PMC9608081

[9] Guo W Lu X Liu Q Zhang T Li P Qiao W . Prognostic value of neutrophil‐to‐lymphocyte ratio and platelet‐to‐lymphocyte ratio for breast cancer patients: an updated meta‐analysis of 17079 individuals. Cancer Med. (2019) 8:4135–48. doi: 10.1002/cam4.2281. PMID: 31197958 PMC6675722

[10] Mohri T Mohri Y Shigemori T Takeuchi K Itoh Y Kato T . Impact of prognostic nutritional index on long-term outcomes in patients with breast cancer. World J Surg Onc. (2016) 14:170. doi: 10.1186/s12957-016-0920-7. PMID: 27349744 PMC4924248

[11] Azab BN Bhatt VR Vonfrolio S Bachir R Rubinshteyn V Alkaied H . Value of the pretreatment albumin to globulin ratio in predicting long-term mortality in breast cancer patients. Am J Surg. (2013) 206:764–70. doi: 10.1016/j.amjsurg.2013.03.007. PMID: 23866764

[12] Liu L Zhang BB Li YZ Huang WJ Niu Y Jia QC . Preoperative glucose-to-lymphocyte ratio predicts survival in cancer. Front Endocrinol. (2024) 15:1284152. doi: 10.3389/fendo.2024.1284152. PMID: 38501103 PMC10946689

[13] Lin F Xia W Chen M Jiang T Guo J Ouyang Y . A prognostic model based on nutritional risk index in operative breast cancer. Nutrients. (2022) 14:3783. doi: 10.3390/nu14183783. PMID: 36145159 PMC9502262

[14] Dirican A Kucukzeybek BB Alacacioglu A Kucukzeybek Y Erten C Varol U . Do the derived neutrophil to lymphocyte ratio and the neutrophil to lymphocyte ratio predict prognosis in breast cancer? Int J Clin Oncol. (2015) 20:70–81. doi: 10.1007/s10147-014-0672-8. PMID: 24532163

[15] Zhang Y Sun Y Zhang Q . Prognostic value of the systemic immune-inflammation index in patients with breast cancer: a meta-analysis. Cancer Cell Int. (2020) 20:224. doi: 10.1186/s12935-020-01308-6. PMID: 32528232 PMC7282128

[16] Zhu M Chen L Kong X Wang X Fang Y Li X . The systemic inflammation response index as an independent predictor of survival in breast cancer patients: a retrospective study. Front Mol Biosci. (2022) 9:856064. doi: 10.3389/fmolb.2022.856064. PMID: 35295846 PMC8918696

[17] Habanjar O Bingula R Decombat C Diab-Assaf M Caldefie-Chezet F Delort L . Crosstalk of inflammatory cytokines within the breast tumor microenvironment. Ijms. (2023) 24:4002. doi: 10.3390/ijms24044002. PMID: 36835413 PMC9964711

[18] Carnevale S Di Ceglie I Grieco G Rigatelli A Bonavita E Jaillon S . Neutrophil diversity in inflammation and cancer. Front Immunol. (2023) 14:1180810. doi: 10.3389/fimmu.2023.1180810. PMID: 37180120 PMC10169606

[19] Gerratana L Basile D Toffoletto B Bulfoni M Zago S Magini A . Biologically driven cut-off definition of lymphocyte ratios in metastatic breast cancer and association with exosomal subpopulations and prognosis. Sci Rep. (2020) 10:7010. doi: 10.1038/s41598-020-63291-2. PMID: 32332763 PMC7181663

[20] De Giorgi U Mego M Scarpi E Giordano A Giuliano M Valero V . Association between circulating tumor cells and peripheral blood monocytes in metastatic breast cancer. Ther Adv In Med Oncol. (2019) 11:1758835919866065. doi: 10.1177/1758835919866065. PMID: 31452692 PMC6696837

[21] Wei C Ai H Mo D Wang P Wei L Liu Z . A nomogram based on inflammation and nutritional biomarkers for predicting the survival of breast cancer patients. Front Endocrinol. (2024) 15:1388861. doi: 10.3389/fendo.2024.1388861. PMID: 39170737 PMC11335604

[22] Zhang S Cheng T . Prognostic and clinicopathological value of systemic inflammation response index (SIRI) in patients with breast cancer: a meta-analysis. Ann Med. (2024) 56:2337729. doi: 10.1080/07853890.2024.2337729. PMID: 38569199 PMC10993763

[23] Gianni C Palleschi M Schepisi G Casadei C Bleve S Merloni F . Circulating inflammatory cells in patients with metastatic breast cancer: implications for treatment. Front Oncol. (2022) 12:882896. doi: 10.3389/fonc.2022.882896. PMID: 36003772 PMC9393759

[24] Chen Y Song Y Du W Gong L Chang H Zou Z . Tumor-associated macrophages: an accomplice in solid tumor progression. J BioMed Sci. (2019) 26:78. doi: 10.1186/s12929-019-0568-z. PMID: 31629410 PMC6800990

[25] Costa A Kieffer Y Scholer-Dahirel A Pelon F Bourachot B Cardon M . Fibroblast heterogeneity and immunosuppressive environment in human breast cancer. Cancer Cell. (2018) 33:463–79. doi: 10.1016/j.ccell.2018.01.011. PMID: 29455927

[26] Singh N Baby D Rajguru JP Patil PB Thakkannavar SS Pujari VB . Inflammation and cancer. Ann Afr Med. (2019) 18:121–6. doi: 10.4103/aam.aam_56_18. PMID: 31417011 PMC6704802

[27] Migita K Takayama T Saeki K Matsumoto S Wakatsuki K Enomoto K . The prognostic nutritional index predicts long-term outcomes of gastric cancer patients independent of tumor stage. Ann Surg Oncol. (2013) 20:2647–54. doi: 10.1245/s10434-013-2926-5. PMID: 23463091

[28] Mountzios G Samantas E Senghas K Zervas E Krisam J Samitas K . Association of the advanced lung cancer inflammation index (ALI) with immune checkpoint inhibitor efficacy in patients with advanced non-small-cell lung cancer. ESMO Open. (2021) 6:100254. doi: 10.1016/j.esmoop.2021.100254. PMID: 34481329 PMC8417333

[29] Jiang H Li B Wu M Wang Q Li Y . Association of the advanced lung cancer inflammation index (ALI) and Gustave Roussy Immune (GRIm) score with immune checkpoint inhibitor efficacy in patients with gastrointestinal and lung cancer. BMC Cancer. (2024) 24:428. doi: 10.1186/s12885-024-12149-1. PMID: 38589844 PMC11000368

[30] Shimada H Fujimoto A Matsuura K Kohyama S Nukui A Ichinose Y . Comprehensive prognostic prediction of metastatic breast cancer treated with eribulin using blood-based parameters and ratio. Mol And Clin Oncol. (2024) 20:15. doi: 10.3892/mco.2024.2713. PMID: 38274088 PMC10809355

[31] Camilleri GM Delrieu L Bouleuc C Pierga JY Cottu P Berger F . Prevalence and survival implications of malnutrition and sarcopenia in metastatic breast cancer: a longitudinal analysis. Clin Nutr. (2024) 43:1710–8. doi: 10.1016/j.clnu.2024.06.014. PMID: 38908032

[32] Spiekerman AM . Proteins used in nutritional assessment. Clin Lab Med. (1993) 13:353–69. doi: 10.1016/s0272-2712(18)30443-8 8319424

[33] Borgoni S Kudryashova KS Burka K de Magalhães JP . Targeting immune dysfunction in aging. Ageing Res Rev. (2021) 70:101410. doi: 10.1016/j.arr.2021.101410. PMID: 34280555

[34] Kourea H Zolota V Scopa C . Targeted pathways in breast cancer: molecular and protein markers guiding therapeutic decisions. Curr Mol Pharmacol. (2015) 7:4–21. doi: 10.2174/187446720701150105170830. PMID: 25563853

[35] Conway JW Braden J Wilmott JS Scolyer RA Long GV Pires da Silva I . The effect of organ-specific tumor microenvironments on response patterns to immunotherapy. Front Immunol. (2022) 13:1030147. doi: 10.3389/fimmu.2022.1030147. PMID: 36466910 PMC9713699

[36] Sharma A Jasrotia S Kumar A . Effects of chemotherapy on the immune system: implications for cancer treatment and patient outcomes. N-s Arch Pharmacol. (2024) 397:2551–66. doi: 10.1007/s00210-023-02781-2. PMID: 37906273

[37] Rashid OM Nagahashi M Ramachandran S Graham L Yamada A Spiegel S . Resection of the primary tumor improves survival in metastatic breast cancer by reducing overall tumor burden. Surgery. (2013) 153:771–8. doi: 10.1016/j.surg.2013.02.002. PMID: 23489938 PMC3664113

[38] Thatishetty AV Agresti N O’Brien CB . Chemotherapy-induced hepatotoxicity. Clin Liver Dis. (2013) 17:671–86. doi: 10.1016/j.cld.2013.07.010. PMID: 24099024

[39] Hadad SM Baker L Quinlan PR Robertson KE Bray SE Thomson G . Histological evaluation of AMPK signalling in primary breast cancer. BMC Cancer. (2009) 9:307. doi: 10.1186/1471-2407-9-307. PMID: 19723334 PMC2744705

