# Supplementary material for: Prognostic evaluation using nutrition-inflammation biomarkers from routine blood tests in metastatic breast cancer: a Boruta algorithm-optimized feature selection study
Source: Front Oncol. 2026 May 21;16:1834427. doi: 10.3389/fonc.2026.1834427 (PMC13233192; doi:10.3389/fonc.2026.1834427)
Supplement: Supplementary file 1 [file DataSheet1.docx]

Table S 1 Calculation formulas for 18 inflammation and nutritional indices.

Each formula is derived from clinical measurements of blood components, albumin, glucose, and anthropometric data.

| **Abbreviation** | **Full Name** | **Calculation Formula** |
| --- | --- | --- |
| **ALI** | Advanced lung cancer inflammation index | Body Mass Index (kg/m^2)*Serum Albumin (g/dl)/Neutrophil to Lymphocyte Ratio |
| **NLR** | Neutrophil to Lymphocyte Ratio | Neutrophils/Lymphocytes |
| **dNLR** | Derived Neutrophil to Lymphocyte Ratio | Neutrophils/(Total White Blood Cell count- Lymphocyte count) |
| **SII** | Systemic Immune-Inflammation Index | Platelet Count*Neutrophil Count/Lymphocyte Count |
| **SIRI** | Systemic Inflammatory Response Index | Monocyte Count*Neutrophil Count/Lymphocyte Count |
| **PLR** | Platelet to Lymphocyte Ratio | Platelets/Lymphocytes |
| **MLR** | Monocyte to Lymphocyte Ratio | Monocyte Count/Lymphocyte Count |
| **NMLR** | Neutrophil and Monocyte to Lymphocyte Ratio | (Monocyte Count+ Neutrophil Count)/Lymphocyte Count |
| **PNI** | Prognostic Nutritional Index | Serum Albumin (g/L) + 5 *Peripheral Blood Lymphocyte Count (×10^9) |
| **GLR** | Glucose to Lymphocyte Ratio | Glucose (mmol/L)/Lymphocytes (×10^9) |
| **AGR** | Albumin to Globulin Ratio | Serum Albumin/Serum Globulin |
| **GNRI** | Geriatric Nutritional Risk Index | 1.489*Serum Albumin (g/L) + 41.7*({Actual Weight/Ideal Weight) |
| **mGNRI** | Modified Geriatric Nutritional Risk Index | 1.489*Serum Albumin (g/L)+ 41.7 *(Actual Weight/Ideal Weight if Actual Weight≤Ideal Weight, otherwise 1) |
| **IBW** | Ideal Body Weight | (Height - 70) * 0.6 |
| **TP** | Total Protein | Actual Test Value |
| **PA** | Prealbumin | Actual Test Value |
| **TRF** | Transferrin | Actual Test Value |
| **ALB** | Albumin | Actual Test Value |

Table S2 Baseline characteristics of patients grouped by AGR.

| **Characteristic** | **AGR-group** | | **p-value** |
| --- | --- | --- | --- |
|  | **< 1.11, N = 38** | **≥ 1.11, N = 125** |  |
| **Age (years),Mean ± SD** | 53 ± 12 | 50 ± 11 | 0.195 |
| **Insurance, n (%)** |  |  | 0.891 |
| No | 12 (31.6%) | 38 (30.4%) |  |
| Yes | 26 (68.4%) | 87 (69.6%) |  |
| **Hypertension, n (%)** |  |  | 0.029 |
| No | 27 (71.1%) | 108 (86.4%) |  |
| Yes | 11 (28.9%) | 17 (13.6%) |  |
| **Diabetes, n (%)** |  |  | 0.682 |
| No | 37 (97.4%) | 118 (94.4%) |  |
| Yes | 1 (2.6%) | 7 (5.6%) |  |
| **Marital status, n (%)** |  |  | 0.265 |
| Married | 33 (86.8%) | 116 (92.8%) |  |
| Unmarried | 3 (7.9%) | 3 (2.4%) |  |
| Widow | 2 (5.3%) | 4 (3.2%) |  |
| Divorced | 0 (0.0%) | 2 (1.6%) |  |
| **Education, n (%)** |  |  | 0.534 |
| Primary School | 9 (23.7%) | 24 (19.2%) |  |
| Middle School | 11 (28.9%) | 32 (25.6%) |  |
| High School | 3 (7.9%) | 10 (8.0%) |  |
| Undergraduate | 2 (5.3%) | 20 (16.0%) |  |
| Others | 13 (34.2%) | 39 (31.2%) |  |
| **Location, n (%)** |  |  | 0.097 |
| Villige | 25 (65.8%) | 63 (50.4%) |  |
| City | 13 (34.2%) | 62 (49.6%) |  |
| **Menstrual status, n (%)** |  |  | 0.262 |
| Postmenopausal | 24 (63.2%) | 66 (52.8%) |  |
| Premenopausal | 14 (36.8%) | 59 (47.2%) |  |
| **Pathological type, n (%)** |  |  | >0.999 |
| Invasive ductal carcinoma | 36 (94.7%) | 116 (92.8%) |  |
| Invasive lobular carcinoma | 1 (2.6%) | 5 (4.0%) |  |
| Others | 1 (2.6%) | 4 (3.2%) |  |
| **Histological grade, n (%)** |  |  | 0.706 |
| 2 | 29 (76.3%) | 99 (79.2%) |  |
| 3 | 9 (23.7%) | 26 (20.8%) |  |
| **Subtype, n (%)** |  |  | 0.330 |
| HR+/HER2- | 23 (60.5%) | 66 (52.8%) |  |
| HR+/HER2+ | 7 (18.4%) | 28 (22.4%) |  |
| HR-/HER2- | 4 (10.5%) | 6 (4.8%) |  |
| HR-/HER2+ | 4 (10.5%) | 25 (20.0%) |  |
| **T stage, n (%)** |  |  | 0.147 |
| T1 | 1 (2.6%) | 7 (5.6%) |  |
| T2 | 7 (18.4%) | 39 (31.2%) |  |
| T3 | 2 (5.3%) | 14 (11.2%) |  |
| T4 | 28 (73.7%) | 65 (52.0%) |  |
| **N stage, n (%)** |  |  | 0.020 |
| N0 | 1 (2.6%) | 14 (11.2%) |  |
| N1 | 10 (26.3%) | 56 (44.8%) |  |
| N2 | 15 (39.5%) | 25 (20.0%) |  |
| N3 | 12 (31.6%) | 30 (24.0%) |  |
| **Metastatic site, n (%)** |  |  | 0.023 |
| Bone | 29 (76.3%) | 65 (52.0%) |  |
| Liver | 5 (13.2%) | 23 (18.4%) |  |
| Lung | 4 (10.5%) | 37 (29.6%) |  |
| **Surgery, n (%)** |  |  | 0.024 |
| No | 28 (73.7%) | 66 (52.8%) |  |
| Yes | 10 (26.3%) | 59 (47.2%) |  |
| **Radiation, n (%)** |  |  | 0.405 |
| No | 34 (89.5%) | 105 (84.0%) |  |
| Yes | 4 (10.5%) | 20 (16.0%) |  |
| **Chemotherapy, n (%)** |  |  | 0.151 |
| No | 16 (42.1%) | 37 (29.6%) |  |
| Yes | 22 (57.9%) | 88 (70.4%) |  |
| **Ki-67,Median (IQR)** | 0.35 (0.20, 0.50) | 0.35 (0.20, 0.50) | 0.895 |
| Globulin （g/L）, Median (IQR) | 36.6 (35.2, 37.6) | 28.7 (26.3, 31.3) | <0.001 |
| Glucose (mmol/L）, Median (IQR) | 4.98 (4.57, 6.49) | 4.80 (4.39, 5.27) | 0.121 |
| **Transferrin (g/L), Median (IQR)** | 2.22 (1.97, 2.47) | 2.42 (2.06, 2.70) | 0.086 |
| **Prealbumin (mg/L), Median (IQR)** | 181 (141, 240) | 225 (202, 266) | <0.001 |
| **White Blood Cell (10^9 /L), Median (IQR)** | 6.77 (5.65, 7.88) | 6.46 (5.42, 7.53) | 0.291 |
| **Platelet (10^9 /L), Median (IQR)** | 291 ± 93 | 274 ± 79 | 0.314 |
| **Monocyte (10^9 /L), Median (IQR)** | 0.44 (0.32, 0.60) | 0.41 (0.34, 0.51) | 0.221 |
| **Total Protein (g/L), Median (IQR)** | 71 ± 7 | 69 ± 6 | 0.092 |
| **Albumin (g/dL), Median (IQR)** | 35.6 ± 4.0 | 39.8 ± 4.0 | <0.001 |
| **Neutrophil (10^9 /L), Median (IQR)** | 4.46 (3.24, 5.64) | 4.01 (3.24, 5.03) | 0.137 |
| **Lymphocyte (10^9 /L), Median (IQR)** | 1.67 (1.38, 2.09) | 1.74 (1.43, 2.26) | 0.543 |
| **BMI, Mean ± SD** | 22.5 ± 3.5 | 23.1 ± 3.2 | 0.415 |

Table S3 Baseline characteristics of patients grouped by SIRI.

| **Characteristic** | **SIRI-group** | | **p-value** |
| --- | --- | --- | --- |
|  | **< 1.7, N = 128** | **≥ 1.7, N = 35** |  |
| **Age (years),Mean ± SD** | 52 ± 12 | 48 ± 9 | 0.058 |
| **Insurance, n (%)** |  |  | 0.350 |
| No | 37 (28.9%) | 13 (37.1%) |  |
| Yes | 91 (71.1%) | 22 (62.9%) |  |
| **Hypertension, n (%)** |  |  | 0.132 |
| No | 109 (85.2%) | 26 (74.3%) |  |
| Yes | 19 (14.8%) | 9 (25.7%) |  |
| **Diabetes, n (%)** |  |  | >0.999 |
| No | 121 (94.5%) | 34 (97.1%) |  |
| Yes | 7 (5.5%) | 1 (2.9%) |  |
| **Marital status, n (%)** |  |  | 0.777 |
| Married | 115 (89.8%) | 34 (97.1%) |  |
| Unmarried | 5 (3.9%) | 1 (2.9%) |  |
| Widow | 6 (4.7%) | 0 (0.0%) |  |
| Divorced | 2 (1.6%) | 0 (0.0%) |  |
| **Education, n (%)** |  |  | 0.135 |
| Primary School | 24 (18.8%) | 9 (25.7%) |  |
| Middle School | 36 (28.1%) | 7 (20.0%) |  |
| High School | 13 (10.2%) | 0 (0.0%) |  |
| Undergraduate | 18 (14.1%) | 4 (11.4%) |  |
| Others | 37 (28.9%) | 15 (42.9%) |  |
| **Location, n (%)** |  |  | 0.236 |
| Villige | 66 (51.6%) | 22 (62.9%) |  |
| City | 62 (48.4%) | 13 (37.1%) |  |
| **Menstrual status, n (%)** |  |  | 0.612 |
| Postmenopausal | 72 (56.3%) | 18 (51.4%) |  |
| Premenopausal | 56 (43.8%) | 17 (48.6%) |  |
| **Pathological type, n (%)** |  |  | >0.999 |
| Invasive ductal carcinoma | 119 (93.0%) | 33 (94.3%) |  |
| Invasive lobular carcinoma | 5 (3.9%) | 1 (2.9%) |  |
| Others | 4 (3.1%) | 1 (2.9%) |  |
| **Histological grade, n (%)** |  |  | 0.038 |
| 2 | 105 (82.0%) | 23 (65.7%) |  |
| 3 | 23 (18.0%) | 12 (34.3%) |  |
| **Subtype, n (%)** |  |  | 0.102 |
| HR+/HER2- | 73 (57.0%) | 16 (45.7%) |  |
| HR+/HER2+ | 29 (22.7%) | 6 (17.1%) |  |
| HR-/HER2- | 5 (3.9%) | 5 (14.3%) |  |
| HR-/HER2+ | 21 (16.4%) | 8 (22.9%) |  |
| **T stage, n (%)** |  |  | 0.568 |
| T1 | 8 (6.3%) | 0 (0.0%) |  |
| T2 | 36 (28.1%) | 10 (28.6%) |  |
| T3 | 13 (10.2%) | 3 (8.6%) |  |
| T4 | 71 (55.5%) | 22 (62.9%) |  |
| **N stage, n (%)** |  |  | 0.378 |
| N0 | 11 (8.6%) | 4 (11.4%) |  |
| N1 | 56 (43.8%) | 10 (28.6%) |  |
| N2 | 29 (22.7%) | 11 (31.4%) |  |
| N3 | 32 (25.0%) | 10 (28.6%) |  |
| **Metastatic site, n (%)** |  |  | 0.282 |
| Bone | 77 (60.2%) | 17 (48.6%) |  |
| Liver | 19 (14.8%) | 9 (25.7%) |  |
| Lung | 32 (25.0%) | 9 (25.7%) |  |
| **Surgery, n (%)** |  |  | 0.142 |
| No | 70 (54.7%) | 24 (68.6%) |  |
| Yes | 58 (45.3%) | 11 (31.4%) |  |
| **Radiation, n (%)** |  |  | 0.091 |
| No | 106 (82.8%) | 33 (94.3%) |  |
| Yes | 22 (17.2%) | 2 (5.7%) |  |
| **Chemotherapy, n (%)** |  |  | 0.575 |
| No | 43 (33.6%) | 10 (28.6%) |  |
| Yes | 85 (66.4%) | 25 (71.4%) |  |
| **Ki-67,Median (IQR)** | 0.34 (0.20, 0.50) | 0.40 (0.25, 0.58) | 0.128 |
| **Globulin （g/L）, Median (IQR)** | 29.7 (27.0, 33.6) | 31.4 (28.0, 36.3) | 0.209 |
| **Glucose (mmol/L）, Median (IQR)** | 4.86 (4.48, 5.45) | 4.73 (4.14, 5.35) | 0.194 |
| **Transferrin (g/L), Median (IQR)** | 2.40 (2.06, 2.66) | 2.37 (1.95, 2.61) | 0.361 |
| **Prealbumin (mg/L), Median (IQR)** | 225 (197, 263) | 212 (141, 238) | 0.006 |
| **White Blood Cell (10^9 /L), Median (IQR)** | 6.22 (5.26, 7.30) | 8.34 (7.29, 9.91) | <0.001 |
| **Platelet (10^9 /L), Median (IQR)** | 263 ± 79 | 330 ± 73 | <0.001 |
| **Monocyte (10^9 /L), Median (IQR)** | 0.39 (0.30, 0.46) | 0.59 (0.50, 0.69) | <0.001 |
| **Total Protein (g/L), Median (IQR)** | 70 ± 6 | 68 ± 7 | 0.150 |
| **Albumin (g/dL), Median (IQR)** | 39.5 ± 3.9 | 36.5 ± 5.2 | 0.002 |
| **Neutrophil (10^9 /L), Median (IQR)** | 3.89 (3.05, 4.39) | 6.10 (5.25, 7.36) | <0.001 |
| **Lymphocyte (10^9 /L), Median (IQR)** | 1.83 (1.48, 2.30) | 1.44 (1.20, 1.84) | <0.001 |
| **BMI, Mean ± SD** | 22.8 ± 3.3 | 23.4 ± 3.3 | 0.325 |

Table S4 Baseline characteristics of patients grouped by ALI.

| **Characteristic** | **ALI_group** | | **p-value** |
| --- | --- | --- | --- |
|  | **< 53.99, N = 122** | **≥ 53.99, N = 41** |  |
| **Age (years),Mean ± SD** | 50 ± 11 | 53 ± 13 | 0.118 |
| **Insurance, n (%)** |  |  | 0.869 |
| No | 37 (30.3%) | 13 (31.7%) |  |
| Yes | 85 (69.7%) | 28 (68.3%) |  |
| **Hypertension, n (%)** |  |  | 0.619 |
| No | 100 (82.0%) | 35 (85.4%) |  |
| Yes | 22 (18.0%) | 6 (14.6%) |  |
| **Diabetes, n (%)** |  |  | 0.110 |
| No | 118 (96.7%) | 37 (90.2%) |  |
| Yes | 4 (3.3%) | 4 (9.8%) |  |
| **Marital status, n (%)** |  |  | 0.707 |
| Married | 112 (91.8%) | 37 (90.2%) |  |
| Unmarried | 4 (3.3%) | 2 (4.9%) |  |
| Widow | 5 (4.1%) | 1 (2.4%) |  |
| Divorced | 1 (0.8%) | 1 (2.4%) |  |
| **Education, n (%)** |  |  | 0.690 |
| Primary School | 26 (21.3%) | 7 (17.1%) |  |
| Middle School | 31 (25.4%) | 12 (29.3%) |  |
| High School | 8 (6.6%) | 5 (12.2%) |  |
| Undergraduate | 16 (13.1%) | 6 (14.6%) |  |
| Others | 41 (33.6%) | 11 (26.8%) |  |
| **Location, n (%)** |  |  | 0.962 |
| Villige | 66 (54.1%) | 22 (53.7%) |  |
| City | 56 (45.9%) | 19 (46.3%) |  |
| **Menstrual status, n (%)** |  |  | 0.223 |
| Postmenopausal | 64 (52.5%) | 26 (63.4%) |  |
| Premenopausal | 58 (47.5%) | 15 (36.6%) |  |
| **Pathological type, n (%)** |  |  | 0.858 |
| Invasive ductal carcinoma | 114 (93.4%) | 38 (92.7%) |  |
| Invasive lobular carcinoma | 4 (3.3%) | 2 (4.9%) |  |
| Others | 4 (3.3%) | 1 (2.4%) |  |
| **Histological grade, n (%)** |  |  | 0.932 |
| 2 | 96 (78.7%) | 32 (78.0%) |  |
| 3 | 26 (21.3%) | 9 (22.0%) |  |
| **Subtype, n (%)** |  |  | 0.586 |
| HR+/HER2- | 68 (55.7%) | 21 (51.2%) |  |
| HR+/HER2+ | 25 (20.5%) | 10 (24.4%) |  |
| HR-/HER2- | 9 (7.4%) | 1 (2.4%) |  |
| HR-/HER2+ | 20 (16.4%) | 9 (22.0%) |  |
| **T stage, n (%)** |  |  | 0.954 |
| T1 | 6 (4.9%) | 2 (4.9%) |  |
| T2 | 33 (27.0%) | 13 (31.7%) |  |
| T3 | 12 (9.8%) | 4 (9.8%) |  |
| T4 | 71 (58.2%) | 22 (53.7%) |  |
| **N stage, n (%)** |  |  | 0.224 |
| N0 | 12 (9.8%) | 3 (7.3%) |  |
| N1 | 46 (37.7%) | 20 (48.8%) |  |
| N2 | 28 (23.0%) | 12 (29.3%) |  |
| N3 | 36 (29.5%) | 6 (14.6%) |  |
| **Metastatic site, n (%)** |  |  | 0.124 |
| Bone | 66 (54.1%) | 28 (68.3%) |  |
| Liver | 25 (20.5%) | 3 (7.3%) |  |
| Lung | 31 (25.4%) | 10 (24.4%) |  |
| **Surgery, n (%)** |  |  | 0.390 |
| No | 68 (55.7%) | 26 (63.4%) |  |
| Yes | 54 (44.3%) | 15 (36.6%) |  |
| **Radiation, n (%)** |  |  | 0.123 |
| No | 101 (82.8%) | 38 (92.7%) |  |
| Yes | 21 (17.2%) | 3 (7.3%) |  |
| **Chemotherapy, n (%)** |  |  | 0.521 |
| No | 84 (68.9%) | 26 (63.4%) |  |
| Yes | 38 (31.1%) | 15 (36.6%) |  |
| **Ki-67,Median (IQR)** | 0.35 (0.20, 0.50) | 0.30 (0.20, 0.40) | 0.365 |
| **Globulin （g/L）, Median (IQR)** | 29.8 (27.0, 33.9) | 30.1 (27.2, 34.2) | 0.922 |
| **Glucose (mmol/L）, Median (IQR)** | 4.76 (4.36, 5.27) | 5.08 (4.50, 5.56) | 0.103 |
| **Transferrin (g/L), Median (IQR)** | 2.44 (2.08, 2.67) | 2.23 (1.91, 2.50) | 0.116 |
| **Prealbumin (mg/L), Median (IQR)** | 221 (176, 260) | 230 (205, 259) | 0.214 |
| **White Blood Cell (10^9 /L), Median (IQR)** | 6.87 (5.67, 7.98) | 5.98 (5.16, 6.89) | 0.003 |
| **Platelet (10^9 /L), Median (IQR)** | 287 ± 84 | 249 ± 71 | 0.006 |
| **Monocyte (10^9 /L), Median (IQR)** | 0.42 (0.34, 0.53) | 0.39 (0.30, 0.48) | 0.080 |
| **Total Protein (g/L), Median (IQR)** | 69 ± 7 | 70 ± 6 | 0.446 |
| **Albumin (g/dL), Median (IQR)** | 38.5 ± 4.7 | 39.7 ± 3.4 | 0.070 |
| **Neutrophil (10^9 /L), Median (IQR)** | 4.33 (3.70, 5.52) | 3.13 (2.39, 3.84) | <0.001 |
| **Lymphocyte (10^9 /L), Median (IQR)** | 1.62 (1.35, 1.94) | 2.11 (1.89, 2.66) | <0.001 |
| **BMI, Mean ± SD** | 22.6 ± 3.2 | 23.9 ± 3.2 | 0.025 |

Table S5 Baseline characteristics of patients grouped by PA.

| **Characteristic** | **PA-group** | | **p-value** |
| --- | --- | --- | --- |
|  | **< 181, N = 37** | **≥ 181, N = 126** |  |
| **Age (years),Mean ± SD** | 51 ± 11 | 51 ± 11 | 0.993 |
| **Insurance, n (%)** |  |  | 0.585 |
| No | 10 (27.0%) | 40 (31.7%) |  |
| Yes | 27 (73.0%) | 86 (68.3%) |  |
| **Hypertension, n (%)** |  |  | 0.861 |
| No | 31 (83.8%) | 104 (82.5%) |  |
| Yes | 6 (16.2%) | 22 (17.5%) |  |
| **Diabetes, n (%)** |  |  | 0.684 |
| No | 36 (97.3%) | 119 (94.4%) |  |
| Yes | 1 (2.7%) | 7 (5.6%) |  |
| **Marital status, n (%)** |  |  | 0.734 |
| Married | 33 (89.2%) | 116 (92.1%) |  |
| Unmarried | 2 (5.4%) | 4 (3.2%) |  |
| Widow | 2 (5.4%) | 4 (3.2%) |  |
| Divorced | 0 (0.0%) | 2 (1.6%) |  |
| **Education, n (%)** |  |  | 0.440 |
| Primary School | 4 (10.8%) | 29 (23.0%) |  |
| Middle School | 11 (29.7%) | 32 (25.4%) |  |
| High School | 4 (10.8%) | 9 (7.1%) |  |
| Undergraduate | 4 (10.8%) | 18 (14.3%) |  |
| Others | 14 (37.8%) | 38 (30.2%) |  |
| **Location, n (%)** |  |  | 0.132 |
| Villige | 24 (64.9%) | 64 (50.8%) |  |
| City | 13 (35.1%) | 62 (49.2%) |  |
| **Menstrual status, n (%)** |  |  | 0.556 |
| Postmenopausal | 22 (59.5%) | 68 (54.0%) |  |
| Premenopausal | 15 (40.5%) | 58 (46.0%) |  |
| **Pathological type, n (%)** |  |  | 0.371 |
| Invasive ductal carcinoma | 33 (89.2%) | 119 (94.4%) |  |
| Invasive lobular carcinoma | 2 (5.4%) | 4 (3.2%) |  |
| Others | 2 (5.4%) | 3 (2.4%) |  |
| **Histological grade, n (%)** |  |  | 0.165 |
| 2 | 26 (70.3%) | 102 (81.0%) |  |
| 3 | 11 (29.7%) | 24 (19.0%) |  |
| **Subtype, n (%)** |  |  | 0.261 |
| HR+/HER2- | 24 (64.9%) | 65 (51.6%) |  |
| HR+/HER2+ | 4 (10.8%) | 31 (24.6%) |  |
| HR-/HER2- | 3 (8.1%) | 7 (5.6%) |  |
| HR-/HER2+ | 6 (16.2%) | 23 (18.3%) |  |
| **T stage, n (%)** |  |  | 0.093 |
| T1 | 1 (2.7%) | 7 (5.6%) |  |
| T2 | 6 (16.2%) | 40 (31.7%) |  |
| T3 | 2 (5.4%) | 14 (11.1%) |  |
| T4 | 28 (75.7%) | 65 (51.6%) |  |
| **N stage, n (%)** |  |  | 0.206 |
| N0 | 3 (8.1%) | 12 (9.5%) |  |
| N1 | 10 (27.0%) | 56 (44.4%) |  |
| N2 | 11 (29.7%) | 29 (23.0%) |  |
| N3 | 13 (35.1%) | 29 (23.0%) |  |
| **Metastatic site, n (%)** |  |  | 0.202 |
| Bone | 26 (70.3%) | 68 (54.0%) |  |
| Liver | 5 (13.5%) | 23 (18.3%) |  |
| Lung | 6 (16.2%) | 35 (27.8%) |  |
| **Surgery, n (%)** |  |  | 0.315 |
| No | 24 (64.9%) | 70 (55.6%) |  |
| Yes | 13 (35.1%) | 56 (44.4%) |  |
| **Radiation, n (%)** |  |  | 0.814 |
| No | 32 (86.5%) | 107 (84.9%) |  |
| Yes | 5 (13.5%) | 19 (15.1%) |  |
| **Chemotherapy, n (%)** |  |  | 0.433 |
| No | 14 (37.8%) | 39 (31.0%) |  |
| Yes | 23 (62.2%) | 87 (69.0%) |  |
| **Ki-67,Median (IQR)** | 0.30 (0.15, 0.50) | 0.35 (0.25, 0.50) | 0.048 |
| **Globulin （g/L）, Median (IQR)** | 31.6 (29.1, 35.7) | 29.7 (26.9, 33.7) | 0.065 |
| **Glucose (mmol/L）, Median (IQR)** | 4.35 (4.13, 4.98) | 4.96 (4.55, 5.50) | <0.001 |
| **Transferrin (g/L), Median (IQR)** | 2.10 (1.83, 2.45) | 2.45 (2.11, 2.67) | 0.004 |
| **White Blood Cell (10^9 /L), Median (IQR)** | 6.89 (5.71, 8.24) | 6.41 (5.46, 7.55) | 0.182 |
| **Platelet (10^9 /L), Median (IQR)** | 289 ± 88 | 274 ± 81 | 0.348 |
| **Monocyte (10^9 /L), Median (IQR)** | 0.45 (0.37, 0.63) | 0.41 (0.31, 0.51) | 0.013 |
| **Total Protein (g/L), Median (IQR)** | 68 ± 8 | 70 ± 6 | 0.115 |
| **Albumin (g/dL), Median (IQR)** | 35.4 ± 5.0 | 39.8 ± 3.6 | <0.001 |
| **Neutrophil (10^9 /L), Median (IQR)** | 4.38 (3.29, 5.74) | 4.07 (3.17, 4.97) | 0.076 |
| **Lymphocyte (10^9 /L), Median (IQR)** | 1.70 (1.35, 2.05) | 1.75 (1.43, 2.23) | 0.472 |
| **BMI, Mean ± SD** | 21.9 ± 3.3 | 23.2 ± 3.2 | 0.038 |

Table S6 Baseline characteristics of patients grouped by MLR.

| **Characteristic** | **MLR_group** | | **p-value** |
| --- | --- | --- | --- |
|  | **< 0.33, N = 131** | **≥ 0.33, N = 32** |  |
| **Age (years),Mean ± SD** | 51 ± 11 | 50 ± 12 | 0.775 |
| **Insurance, n (%)** |  |  | 0.230 |
| No | 43 (32.8%) | 7 (21.9%) |  |
| Yes | 88 (67.2%) | 25 (78.1%) |  |
| **Hypertension, n (%)** |  |  | 0.192 |
| No | 111 (84.7%) | 24 (75.0%) |  |
| Yes | 20 (15.3%) | 8 (25.0%) |  |
| **Diabetes, n (%)** |  |  | 0.656 |
| No | 125 (95.4%) | 30 (93.8%) |  |
| Yes | 6 (4.6%) | 2 (6.3%) |  |
| **Marital status, n (%)** |  |  | >0.999 |
| Married | 119 (90.8%) | 30 (93.8%) |  |
| Unmarried | 5 (3.8%) | 1 (3.1%) |  |
| Widow | 5 (3.8%) | 1 (3.1%) |  |
| Divorced | 2 (1.5%) | 0 (0.0%) |  |
| **Education, n (%)** |  |  | 0.856 |
| Primary School | 27 (20.6%) | 6 (18.8%) |  |
| Middle School | 35 (26.7%) | 8 (25.0%) |  |
| High School | 11 (8.4%) | 2 (6.3%) |  |
| Undergraduate | 19 (14.5%) | 3 (9.4%) |  |
| Others | 39 (29.8%) | 13 (40.6%) |  |
| **Location, n (%)** |  |  | 0.496 |
| Villige | 69 (52.7%) | 19 (59.4%) |  |
| City | 62 (47.3%) | 13 (40.6%) |  |
| **Menstrual status, n (%)** |  |  | 0.509 |
| Postmenopausal | 74 (56.5%) | 16 (50.0%) |  |
| Premenopausal | 57 (43.5%) | 16 (50.0%) |  |
| **Pathological type, n (%)** |  |  | 0.490 |
| Invasive ductal carcinoma | 122 (93.1%) | 30 (93.8%) |  |
| Invasive lobular carcinoma | 4 (3.1%) | 2 (6.3%) |  |
| Others | 5 (3.8%) | 0 (0.0%) |  |
| **Histological grade, n (%)** |  |  | 0.048 |
| 2 | 107 (81.7%) | 21 (65.6%) |  |
| 3 | 24 (18.3%) | 11 (34.4%) |  |
| **Subtype, n (%)** |  |  | 0.022 |
| HR+/HER2- | 75 (57.3%) | 14 (43.8%) |  |
| HR+/HER2+ | 29 (22.1%) | 6 (18.8%) |  |
| HR-/HER2- | 4 (3.1%) | 6 (18.8%) |  |
| HR-/HER2+ | 23 (17.6%) | 6 (18.8%) |  |
| **T stage, n (%)** |  |  | 0.489 |
| T1 | 8 (6.1%) | 0 (0.0%) |  |
| T2 | 37 (28.2%) | 9 (28.1%) |  |
| T3 | 14 (10.7%) | 2 (6.3%) |  |
| T4 | 72 (55.0%) | 21 (65.6%) |  |
| **N stage, n (%)** |  |  | 0.391 |
| N0 | 11 (8.4%) | 4 (12.5%) |  |
| N1 | 57 (43.5%) | 9 (28.1%) |  |
| N2 | 31 (23.7%) | 9 (28.1%) |  |
| N3 | 32 (24.4%) | 10 (31.3%) |  |
| **Metastatic site, n (%)** |  |  | 0.120 |
| Bone | 80 (61.1%) | 14 (43.8%) |  |
| Liver | 19 (14.5%) | 9 (28.1%) |  |
| Lung | 32 (24.4%) | 9 (28.1%) |  |
| **Surgery, n (%)** |  |  | 0.311 |
| No | 73 (55.7%) | 21 (65.6%) |  |
| Yes | 58 (44.3%) | 11 (34.4%) |  |
| **Radiation, n (%)** |  |  | 0.417 |
| No | 110 (84.0%) | 29 (90.6%) |  |
| Yes | 21 (16.0%) | 3 (9.4%) |  |
| **Chemotherapy, n (%)** |  |  | 0.555 |
| No | 44 (33.6%) | 9 (28.1%) |  |
| Yes | 87 (66.4%) | 23 (71.9%) |  |
| **Ki-67,Median (IQR)** | 0.30 (0.20, 0.50) | 0.40 (0.29, 0.51) | 0.117 |
| **Globulin （g/L）, Median (IQR)** | 29.7 (27.0, 33.7) | 30.2 (27.5, 36.2) | 0.457 |
| **Glucose (mmol/L）, Median (IQR)** | 4.85 (4.49, 5.44) | 4.68 (4.21, 5.63) | 0.240 |
| **Transferrin (g/L), Median (IQR)** | 2.40 (2.05, 2.69) | 2.41 (1.97, 2.58) | 0.512 |
| **Prealbumin (mg/L), Median (IQR)** | 224 (195, 264) | 212 (150, 240) | 0.049 |
| **White Blood Cell (10^9 /L), Median (IQR)** | 6.38 (5.37, 7.47) | 7.45 (6.03, 8.83) | 0.011 |
| **Platelet (10^9 /L), Median (IQR)** | 273 ± 83 | 294 ± 80 | 0.201 |
| **Monocyte (10^9 /L), Median (IQR)** | 0.39 (0.30, 0.48) | 0.60 (0.48, 0.69) | <0.001 |
| **Total Protein (g/L), Median (IQR)** | 70 ± 6 | 68 ± 7 | 0.192 |
| **Albumin (g/dL), Median (IQR)** | 39.3 ± 4.3 | 37.0 ± 4.5 | 0.013 |
| **Neutrophil (10^9 /L), Median (IQR)** | 4.01 (3.13, 4.82) | 5.33 (4.27, 6.24) | <0.001 |
| **Lymphocyte (10^9 /L), Median (IQR)** | 1.85 (1.53, 2.31) | 1.38 (1.07, 1.61) | <0.001 |
| **BMI, Mean ± SD** | 23.0 ± 3.2 | 22.9 ± 3.7 | 0.949 |

Table S7. Bootstrap internal validation of the exploratory combined model and key individual biomarkers.

| **Model** | **Metric** | **Apparent** | **Mean optimism** | **Optimism-corrected** |
| --- | --- | --- | --- | --- |
| **Combined model** | C-index | 0.685 | 0.017 | 0.668 |
| **Combined model** | AUC 12 months | 0.766 | 0.015 | 0.751 |
| **Combined model** | AUC 36 months | 0.668 | 0.023 | 0.645 |
| **Combined model** | AUC 60 months | 0.66 | 0.036 | 0.624 |
| **MLR** | C-index | 0.645 | 0 | 0.645 |
| **MLR** | AUC 12 months | 0.747 | 0 | 0.747 |
| **MLR** | AUC 36 months | 0.592 | 0 | 0.592 |
| **MLR** | AUC 60 months | 0.626 | 0.001 | 0.625 |
| **SIRI** | C-index | 0.651 | 0.002 | 0.649 |
| **SIRI** | AUC 12 months | 0.756 | 0.003 | 0.753 |
| **SIRI** | AUC 36 months | 0.618 | 0.003 | 0.614 |
| **SIRI** | AUC 60 months | 0.544 | 0.003 | 0.541 |
| **ALI** | C-index | 0.634 | 0.001 | 0.633 |
| **ALI** | AUC 12 months | 0.721 | 0 | 0.721 |
| **ALI** | AUC 36 months | 0.628 | 0.001 | 0.626 |
| **ALI** | AUC 60 months | 0.598 | 0.004 | 0.594 |
| **AGR** | C-index | 0.647 | 0.003 | 0.645 |
| **AGR** | AUC 12 months | 0.671 | 0.004 | 0.667 |
| **AGR** | AUC 36 months | 0.658 | 0.004 | 0.654 |
| **AGR** | AUC 60 months | 0.584 | 0.008 | 0.577 |
| **PA** | C-index | 0.614 | 0.001 | 0.613 |
| **PA** | AUC 12 months | 0.651 | 0.004 | 0.646 |
| **PA** | AUC 36 months | 0.606 | 0.001 | 0.604 |
| **PA** | AUC 60 months | 0.554 | 0 | 0.554 |

Table S8. Association between the MLR–ALI risk stratification system and overall survival.

| **Risk group** | **Definition** | **N** | **Events** | **HR (95% CI)** | **p-value** |
| --- | --- | --- | --- | --- | --- |
| Low risk | MLR < 0.33 and ALI ≥ 53.99 | 40 | 5 | Reference | — |
| Intermediate risk | MLR ≥ 0.33 or ALI < 53.99 | 92 | 36 | 3.081 (1.209–7.853) | 0.018 |
| High risk | MLR ≥ 0.33 and ALI < 53.99 | 31 | 20 | 7.158 (2.683–19.095) | <0.001 |

HRs were estimated using Cox proportional hazards regression, with the low-risk group as the reference group. The log-rank p-value for Kaplan–Meier survival comparison among the three groups was 1.98 × 10⁻⁵.


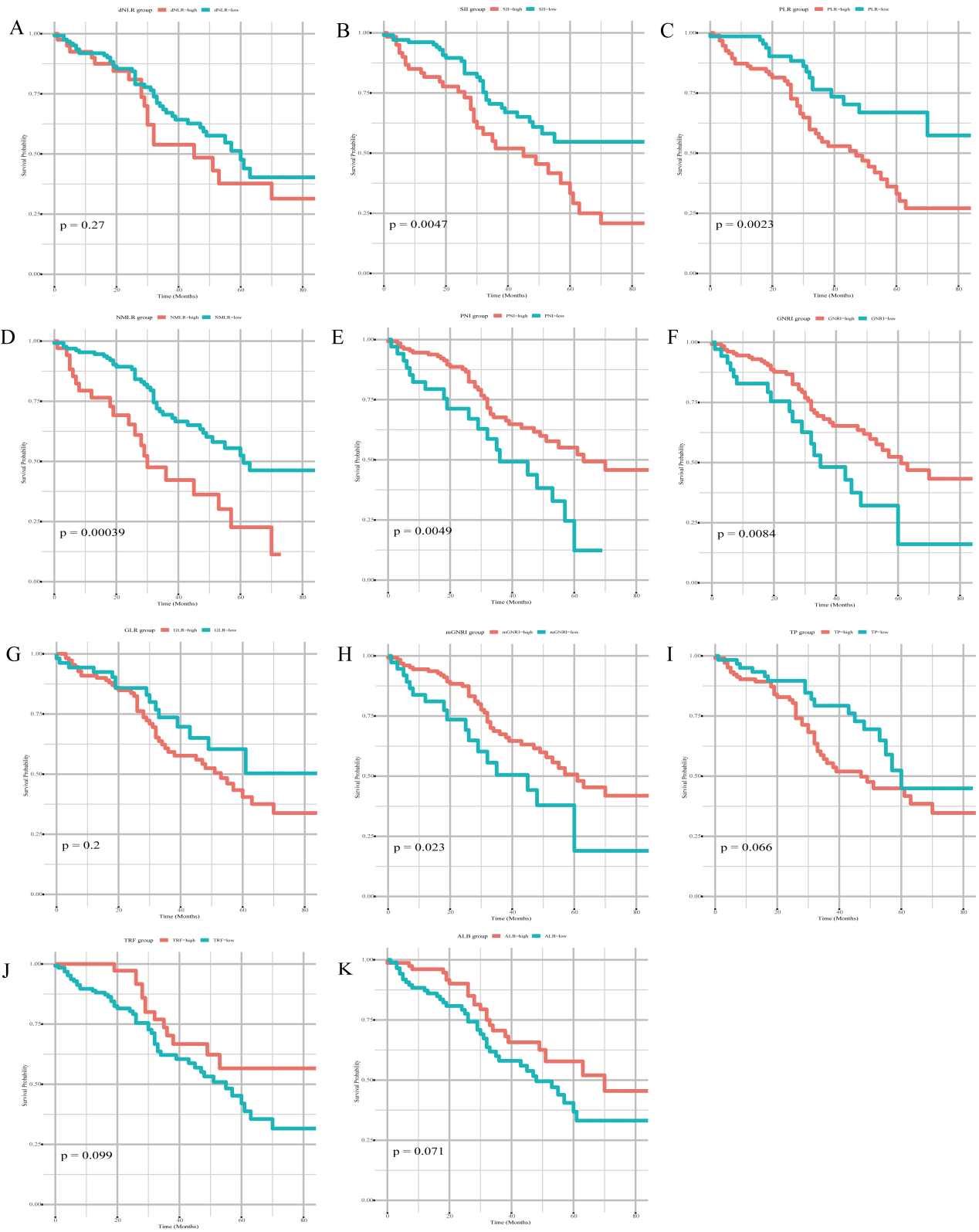


Figure S1. Kaplan–Meier survival curves for overall survival in patients with metastatic breast cancer stratified by additional nutrition- and inflammation-related indicators.

(A) dNLR, (B) SII, (C) PLR, (D) NMLR, (E) PNI, (F) GNRI, (G) GLR, (H) mGNRI, (I) TP, (J) TRF, and (K) ALB.

dNLR, derived neutrophil-to-lymphocyte ratio; SII, systemic immune-inflammation index; PLR, platelet-to-lymphocyte ratio; NMLR, neutrophil-and-monocyte-to-lymphocyte ratio; PNI, prognostic nutritional index; GNRI, geriatric nutritional risk index; GLR, glucose-to-lymphocyte ratio; mGNRI, modified geriatric nutritional risk index; TP, total protein; TRF, transferrin; ALB, albumin.


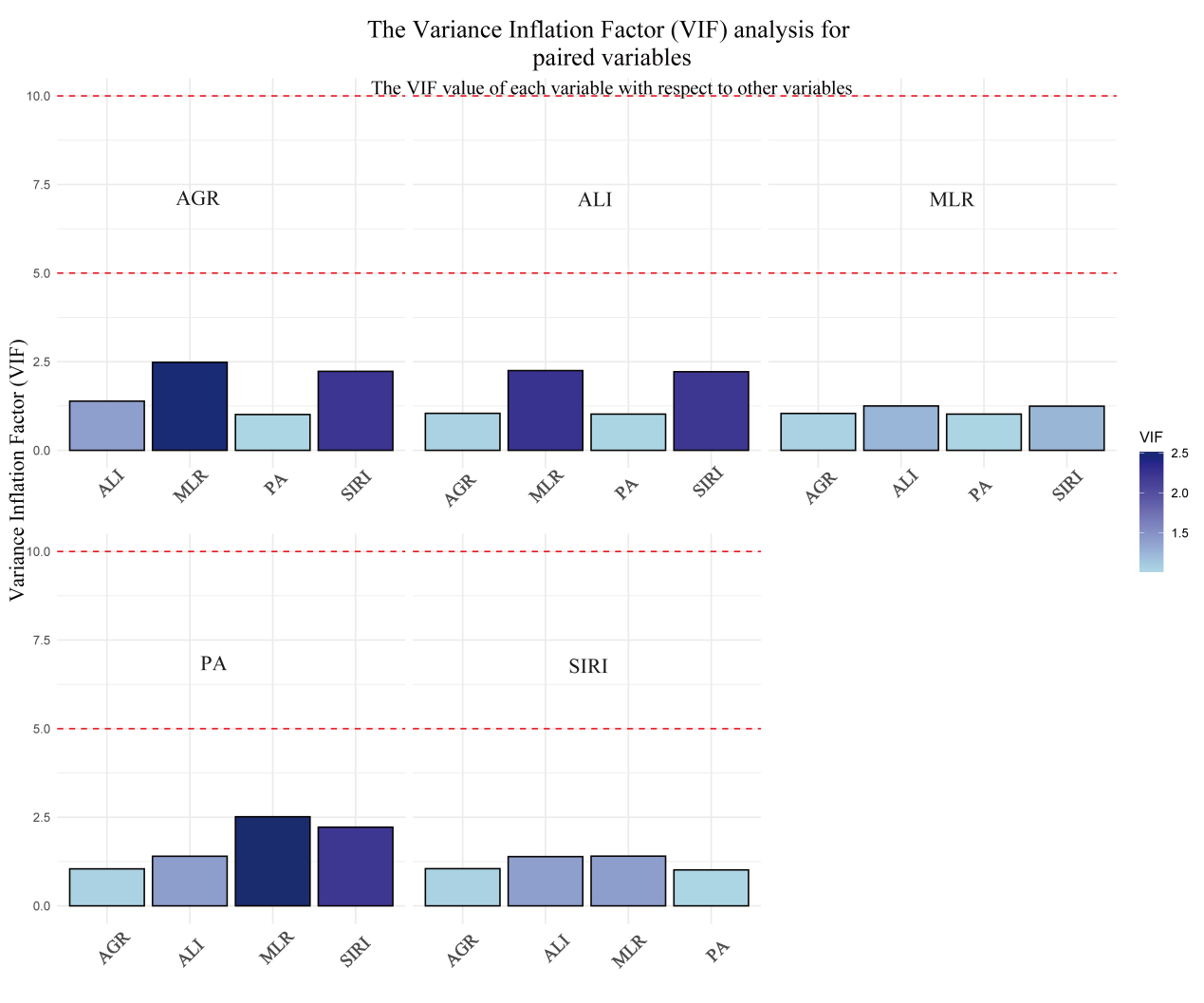


Figure S2. Variance inflation factor analysis of selected prognostic indicators.

The plot shows the variance inflation factor values for ALI, SIRI, MLR, AGR, and PA, which were selected by the Boruta algorithm. All variance inflation factor values were below the commonly used threshold, indicating no substantial multicollinearity among the selected indicators.

ALI, advanced lung cancer inflammation index; SIRI, systemic inflammatory response index; MLR, monocyte-to-lymphocyte ratio; AGR, albumin-to-globulin ratio; PA, prealbumin.


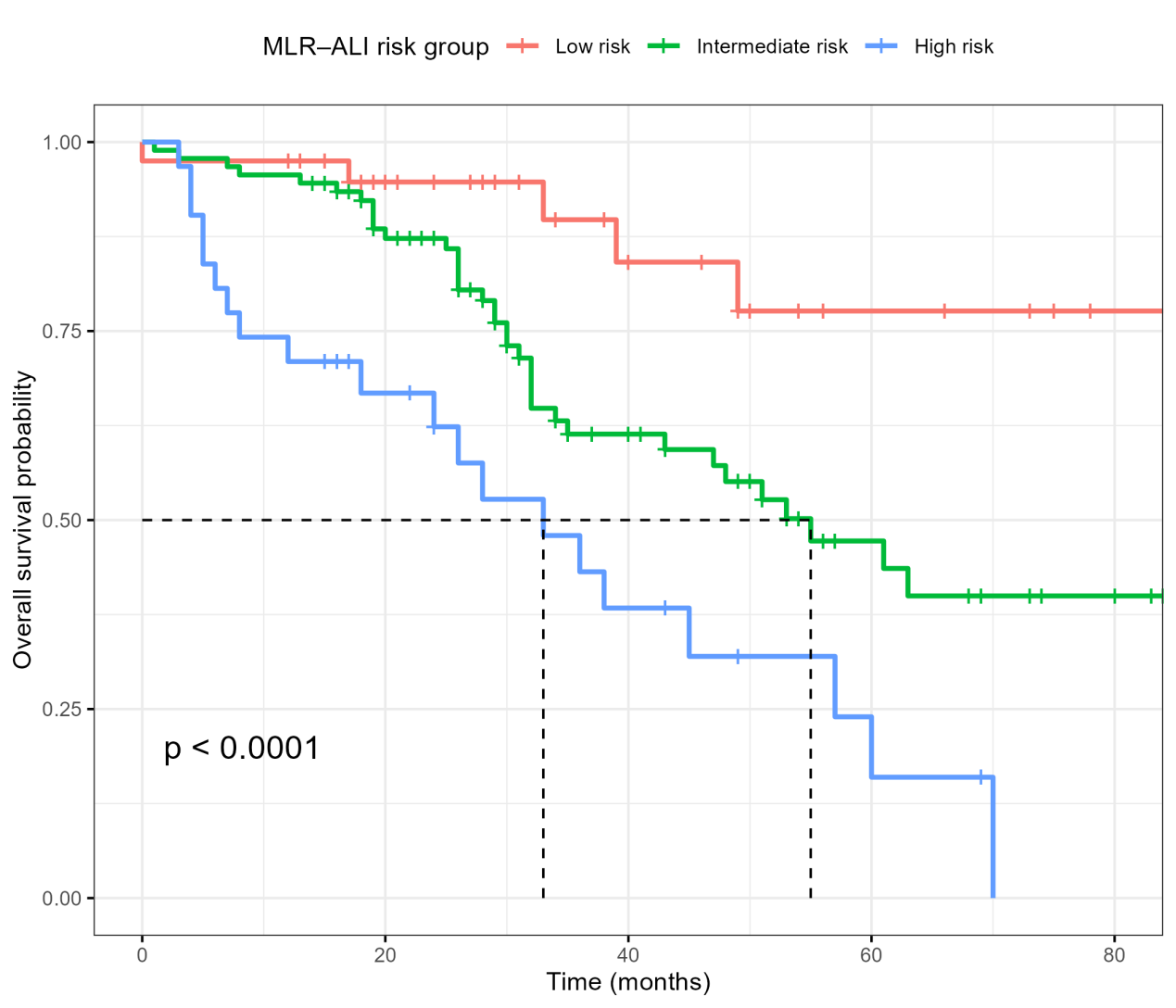


Figure S3. Kaplan–Meier survival curves according to the MLR–ALI risk stratification system.

Kaplan–Meier survival curves for overall survival according to the MLR–ALI risk stratification system. Patients were classified into low-risk, intermediate-risk, and high-risk groups based on the combined status of MLR and ALI. Low risk was defined as MLR < 0.33 and ALI ≥ 53.99; intermediate risk was defined as either MLR ≥ 0.33 or ALI < 53.99; and high risk was defined as MLR ≥ 0.33 and ALI < 53.99. MLR, monocyte-to-lymphocyte ratio; ALI, advanced lung cancer inflammation index.
